# A novel intergenic enhancer that regulates *Bdnf* expression in developing cortical neurons

**DOI:** 10.1016/j.isci.2022.105695

**Published:** 2022-12-01

**Authors:** Emily Brookes, Braulio Martinez De La Cruz, Paraskevi Boulasiki, Ho Yu Alan Au, Wazeer Varsally, Christopher Barrington, Suzana Hadjur, Antonella Riccio

**Affiliations:** 1 Laboratory for Molecular Cell Biology, University College London, Gower Street, London WC1E 6BT, UK; 2 Research Department of Cancer Biology, University College London, Paul O’Gorman Building, 72 Huntley Street, London WC1E 6BT, UK

**Keywords:** Biological sciences, Neuroscience, Molecular neuroscience, Developmental neuroscience

## Abstract

Brain-derived neurotrophic factor (BDNF) promotes neuronal differentiation and survival and is implicated in the pathogenesis of many neurological disorders. Here, we identified a novel intergenic enhancer located 170 kb from the *Bdnf* gene, which promotes the expression of *Bdnf* transcript variants during mouse neuronal differentiation and activity. Following *Bdnf* activation, enhancer-promoter contacts increase, and the region moves away from the repressive nuclear periphery. *Bdnf* enhancer activity is necessary for neuronal clustering and dendritogenesis *in vitro*, and for cortical development *in vivo*. Our findings provide the first evidence of a regulatory mechanism whereby the activation of a distal enhancer promotes *Bdnf* expression during brain development.

## Highlights

• We identify a novel intergenic enhancer, *Bdnf* ^*Enh170*^*,* that loops to the *Bdnf* gene
• *Bdnf*^*Enh170*^ upregulates *Bdnf* mRNAs during neuronal differentiation and activity
• *Bdnf*^*Enh170*^ activity is required for dendritogenesis in cultured neurons
• *Bdnf*^*Enh170*^ activity is necessary for mouse cortical development *in vivo*

## Graphical abstract

**Figure:**
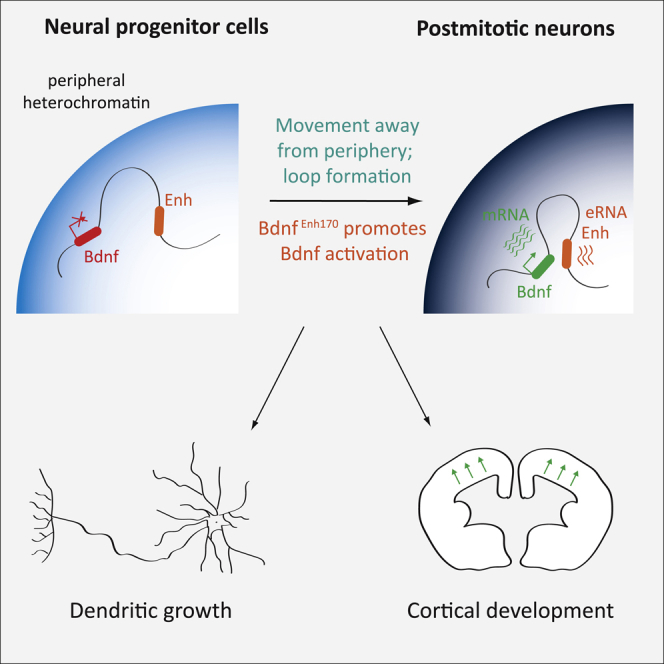

## Introduction

The *brain-derived neurotrophic factor (BDNF)* gene encodes a neurotrophin with critical roles in brain development and functions, ranging from neuronal survival and differentiation during early development, to long-term potentiation and synaptic plasticity in the adult brain.^1^^,^^2^ Reduced BDNF expression has been implicated in a host of neurological diseases, including neuropsychiatric disorders such as schizophrenia,^3^ stress^4^ and depression^5^; neurodegenerative diseases including Huntington’s^6^^,^^7^ and Alzheimer’s disease^8^; and neurodevelopmental disorders such as Rett syndrome^9^ and attention deficit hyperactivity disorder.^10^ Conversely, enhanced BDNF expression is linked to the neuroprotective effects of environmental enrichment,^11^^,^^12^ exercise,^13^^,^^14^ and anti-depressants.^2^^,^^15^ Given the myriad functions identified for BDNF, understanding the regulation of the *BDNF* gene in neurons during brain development and disease is of paramount importance.

The rodent and human structure of the *BDNF* gene is complex, consisting of multiple 5′ exons, each containing its own promoter and 5′ untranslated region (5′UTR), that are alternatively spliced to a universal coding exon^16^^,^^17^^,^^18^ (Figure S1A). Despite being translated into identical proteins, *Bdnf* mRNA variants exhibit specific expression patterns and physiological effects.^19^^,^^20^^,^^21^^,^^22^^,^^23^^,^^24^^,^^25^^,^^26^ For example, disruption of exon I or II, but not IV or VI, enhances male aggression^19^ and impairs female maternal care.^20^ Our current understanding of *Bdnf* transcriptional control is mostly centered on the distinct role of each promoter, however its regulation through distal elements remains unclear.

Enhancers are short regions of regulatory DNA, whose activity promotes the expression of their target gene(s).^27^ Combinations of enhancer elements confer spatially and temporally regulated gene expression profiles.^28^ In linear chromosomal distance, enhancers are often located far from the genes that they control, although within the three dimensional (3D) nuclear space they become proximal through enhancer-promoter looping.^29^ Enhancer-promoter proximity can be critical for appropriate gene expression and is supported by the genome architecture of the region.^30^^,^^31^^,^^32^^,^^33^^,^^34^ Interactions can occur in the context of topologically associated domains (TADs), which are megabase-sized regions of DNA that interact more frequently within themselves than with the surrounding regions.^35^ Genome topology and gene activation is also affected by nuclear compartmentalization, and the position of the gene with respect to nuclear landmarks is important. Putative enhancers for *Bdnf* have been identified based on 3D proximity to the gene and H3K27ac occupancy.^36^ An intronic enhancer regulating both basal and stimulus-dependent expression of *Bdnf* was recently found for transcripts expressed from promoters I-III.^37^

Here, we identify a novel enhancer region that is critical for *Bdnf* expression during neuronal differentiation, dendritic branching, and cortical development. We show that the *Bdnf* gene is located in a previously undescribed sub-TAD, and that the gene is repositioned away from the nuclear periphery during neuronal differentiation. Together, our results identify a mechanism of regulation that is implicated in *Bdnf* expression during neurodevelopmental processes and possibly neurological disorders.

## Results

### Nuclear relocation of the activated *Bdnf* gene during neuronal differentiation

**Figure 1:**
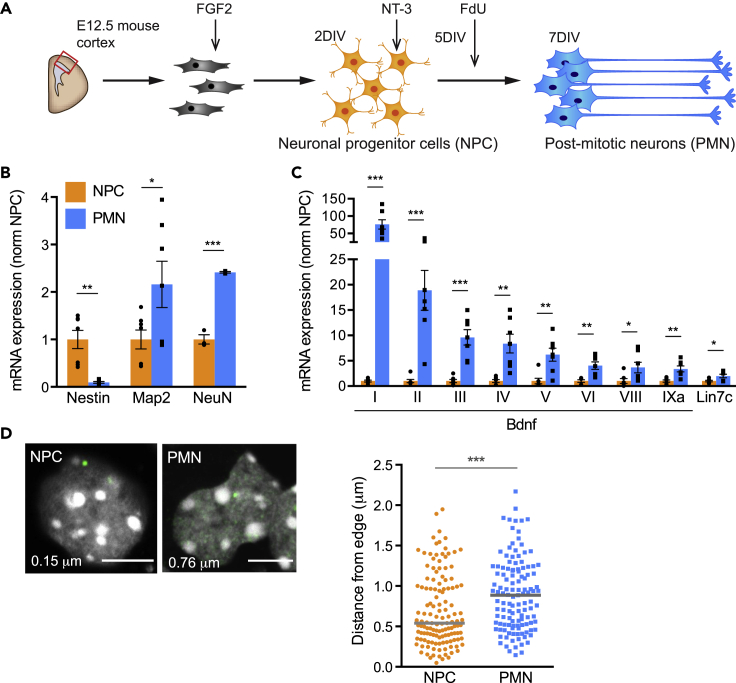
Expression of *Bdnf* isoforms increases over embryonic cortical neuron differentiation concomitant with movement of the gene locus away from the nuclear periphery (A) Schematic of neuronal precursor cell (NPC) differentiation into post-mitotic neurons (PMN). E12.5, embryonic day 12.5. FGF2, fibroblast growth factor 2. NT-3, neurotrophin-3. FdU, 5-fluoro-2′-deoxyuridine. DIV, days *in vitro*. (B) Expression profile of an NPC-marker, Nestin, and neuronal markers, Map2 and NeuN, in NPCs and PMNs, assessed by qRT-PCR and normalized to NPC. Bars represent mean ± SEM; points show results from different biological replicates . ∗p< 0.05, ∗∗p< 0.01, ∗∗∗p< 0.001; unpaired t-test (two-tailed). Nestin p = 0.0005, *t* = 4.707, n = 7, *df* = 12; Map2 p = 0.0481, *t* = 2.201, n = 7,*df* = 12; NeuN p = 0.0001, *t* = 14.15, n = 3, *df* = 4. (C) Expression of *Bdnf* variants and downstream gene *Lin7c* during differentiation, assessed by qRT-PCR and normalized to NPC levels. Bars represent mean ± SEM; points show results from different biological replicates (n = 7, *df* = 12). ∗p< 0.05, ∗∗p< 0.01, ∗∗∗p< 0.001, ∗∗∗∗p< 0.0001, unpaired t-test (two-tailed). Exon I p = 0.0001, *t* = 5.627; Exon II p = 0.0007, *t* = 4.549; Exon III p = 0.0001, *t* = 5.614; Exon IV p = 0.0019, *t* = 3.972; Exon V p = 0.0029, *t* = 3.734; Exon VI p = 0.0021, *t* = 3.899; Exon VIII p = 0.0397, *t* = 2.307; Exon IXa p = 0.0023, *t* = 3.846; Lin7c p = 0.0248, *t* = 2.565. (D) Relocation of the *Bdnf* gene during neuronal development assessed by DNA-FISH combined with measurements of the distance of the signal from the closest edge of the nucleus. Left panel; representative confocal sections of DNA-FISH showing nuclear localization of *Bdnf* loci (green) in NPCs and PMNs. Nuclei were stained with DAPI (gray). For each image, the distance between the center of the FISH signal and the edge of the nucleus is indicated. Scale bars, 5 μm. Right panel; scatter dot plot of the distribution of the distance between *Bdnf* locus and the edge of the DAPI staining. Solid gray lines denote medians. ∗∗∗∗p = 0.0002, Mann-Whitney test (two-tailed). n = 133 (NPC), 123 (PMN) foci across 4 biological replicates. See also Figure S1.

To study the regulation of the *Bdnf* gene during neuronal differentiation, we used a model system previously established in the laboratory.^38^ Neurons were dissected from E12.5 mouse cortices and cultured with fibroblast growth factor 2 (FGF2) for 2 days *in vitro* (DIV) to generate a homogeneous population of neuronal progenitor cells (NPCs) (Figure 1A). NPCs were differentiated into neurons by adding neurotrophin-3 (NT-3) and the anti-mitotic agent 5-fluoro-2′-deoxyuridine (FdU) to remove remaining proliferating cells; post-mitotic neurons (PMN) were harvested after 7 DIV. Expression analysis of the NPC-marker *Nestin* and the neuronal markers *Map2* and *NeuN* were performed to assess neuronal differentiation (Figure 1B). The expression of *Bdnf* isoforms was analyzed by quantitative reverse transcription PCR (qRT-PCR) with a reverse primer complementary to universal exon IX and forward primers matching each 5′UTR (Figure S1A). The expression of all *Bdnf* isoforms increased during the differentiation of NPCs to PMNs, with the exon I-containing isoform showing the most substantial increase (Figure 1C). *Lin7c*, a gene located downstream of *Bdnf* that is expressed in neurons and regulates postsynaptic density,^39^ also increased during neuronal differentiation (Figure 1C).

To understand the mechanisms that facilitate this striking increase in *Bdnf* expression, we first investigated the 3D nuclear position of the *Bdnf* gene. The nucleus is highly organized, with the heterochromatin-enriched nuclear periphery providing an environment suitable to maintain transcriptional repression.^35^ Movement away from the lamina is therefore often concomitant with either gene expression or increased competency for later expression.^40^ The *Bdnf* gene relocates from the nuclear periphery to the nuclear interior in response to kainate-induced seizures in the adult brain.^41^ To study whether this also occurs during neuronal differentiation, DNA fluorescence *in situ* hybridization (FISH) was performed on NPCs and PMNs using a BAC (Bacterial Artificial Chromosome) spanning the *Bdnf* locus. When the distance of *Bdnf* from the edge of the nucleus stained with 4′,6-diamidino-2-phenylindole (DAPI) was measured, a significant movement of the locus away from the nuclear periphery into the more transcriptionally permissive nuclear interior was observed (Figure 1D).

### *Bdnf* loops to a downstream intergenic regulatory site in neurons

In the nucleus, the genome is arranged into self-interacting TADs in which DNA sequences contact each other more frequently.^42^^,^^43^ Strengthening of intra-TAD and depletion of inter-TAD contacts have been observed during neuronal development, and new TAD boundaries form near developmentally regulated genes as they become transcriptionally active.^44^ To study the TAD boundaries that regulate *Bdnf* gene interactions, we analyzed high-resolution HiC data of mouse embryonic stem cells (ESCs) differentiated into NPCs and cortical neurons.^44^ We discovered a sub-TAD encompassing the *Bdnf* gene and a downstream, gene-free region adjacent to the closest gene *Lin7c* (Figure S1B). The sub-TAD falls at the 3′ end of a larger TAD and, despite the dramatic difference in *Bdnf* expression, its contact frequencies appeared similar in NPCs and cortical neurons (Figure S1B).

CTCF (CCCTC-binding factor) and cohesin are key regulators of TAD boundaries,^45^^,^^46^ and are known to bind to the *Bdnf* locus at promoter IV and intron 7 in mouse neurons.^47^ Loss of either CTCF or cohesin compromises *Bdnf* transcription from promoter IV, increasing repressive histone modifications.^47^ Analysis of published CTCF Chromatin Immunoprecipitation with sequencing (ChIP-seq) data^44^ identified peaks in the *Bdnf* and *Lin7c* genes in both NPCs and cortical neurons at sites coinciding with the sub-TAD boundaries (Figure S1B). CTCF binding site 1 spans *Bdnf* exon II, but the highest enrichment of CTCF was observed at *Bdnf* binding site 2, located on the downstream part of exon VII and extending into the intron (Figures S1B and S1C). CTCF binds within *Lin7c* at exon IV. ChIP-qPCR for the cohesin subunit Rad21 confirmed cohesin binding to *Bdnf* CTCF site 2 in primary NPCs and PMNs (Figure S1C). In PMNs, low levels of Rad21 enrichment were seen at *Bdnf* site 1 and at the *Lin7c* site, which were significantly above the IgG control (Figure S1C). These data indicate that during neuronal development *Bdnf* and *Lin7c* co-occupy a sub-TAD with CTCF-positive, cohesin-positive boundaries.

**Figure 2:**
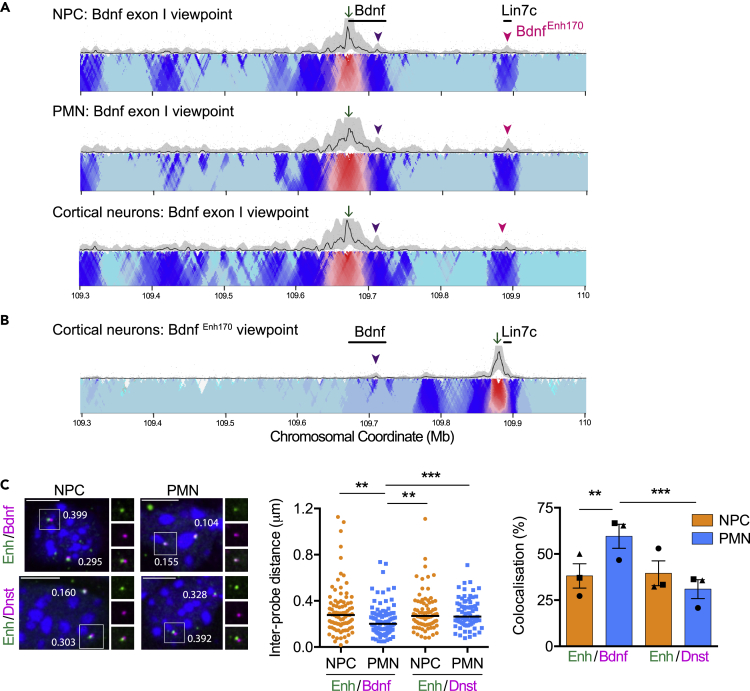
*Bdnf* forms a chromatin loop with a distal interacting site (A and B) Contact profiles from 4C-seq experiments in neuronal progenitor cells (NPC), postmitotic neurons (PMN) and cortical neurons from exon I (A) and *Bdnf*^*Enh170*^ (B) viewpoints (green arrow). Interactions to an intragenic site (purple arrowhead) and to *Bdnf*^*Enh170*^ (pink arrowhead) are indicated. Each image shows a representative 4C-seq experiment (from n = 2) represented by the median normalized 4C-seq coverage in a sliding window of 5 kb (top) and a multi-scale domainogram indicating normalized mean coverage in windows ranging between 2 and 50 kb. (C) Double DNA-FISH of a probe spanning *Bdnf*^*Enh170*^ (Enh) with probes spanning either the *Bdnf* gene (Bdnf) or an equidistant region downstream (Dnst). Left panel, representative maximal intensity projections of double DNA-FISH in NPCs and PMNs. Nuclei were counterstained with DAPI (blue). Scale bar, 5 μm. Middle panel, scatter dot plot of inter-probe distance measurements in NPC (orange) and PMN (blue) cells. Solid lines denote medians. n = 87 (Enh/Bdnf-NPC), 98 (Enh/Bdnf-PMN), 78 (Enh/Dnst-NPC), 74 (Enh/Dnst-PMN) foci across 3 biological replicates. Probe labeling denoted in colored font. ∗∗p< 0.01, ∗∗∗p< 0.001, one-way ANOVA (two-tailed; p< 0.0001) with Dunn’s multiple comparisons: Enh/Bdnf-NPC versus Enh/Bdnf-PMN p = 0.0023; Enh/Bdnf-PMN versus Enh/Dnst-NPC p = 0.0022; Enh/Bdnf-PMN versus Enh/Dnst-PMN p = 0.0008. Right panel, co-localization (defined as an inter-probe distance of 225 nm or less) of FISH signals in double DNA-FISH experiments performed in NPCs and PMNs. Bars represent mean ± SEM, and points show results from different biological replicates (n = 3). ∗∗p< 0.01, ∗∗∗p< 0.001, Fisher’s exact test (two-tailed). Enh/Bdnf-NPC versus Enh/Bdnf-PMN p = 0.0051; Enh/Bdnf-PMN versus Enh/Dnst-PMN p = 0.0002. See also Figure S2.

Sub-TAD level loops often reflect contacts occurring between gene promoters and enhancers,^35^ so we reasoned that enhancers for the *Bdnf* gene could be found within the sub-TAD identified here. To this end, we performed 4C-seq, a technique that identifies chromatin regions making contact with a specific ‘viewpoint’ sequence.^48^ A viewpoint designed at *Bdnf* promoter I identified two regions of interaction in NPCs and PMNs, and in cortical neurons (Figure 2A). The first interaction site is internal to the *Bdnf* gene, around exon VIII. The second is an intergenic region located around 170 kb downstream of *Bdnf* and around 5 kb upstream of the *Lin7c* gene (a distal interacting site, hereby referred to as *Bdnf* ^*Enh170*^; Figure 2A). The profile appeared the same irrespective of the *Bdnf* expression levels in the cell type (Figure 2A), consistent with the HiC analysis (Figure S1B).

To confirm *Bdnf* interaction with the distal interacting site, we designed a viewpoint spanning *Bdnf* ^*Enh170*^ and performed 4C-seq in cortical neuronss. We identified the reciprocal interaction from *Bdnf* ^*Enh170*^ to *Bdnf*, with a peak at exon VIII, suggesting that this site anchors the loop (Figure 2B). Interactions were negligible from *Bdnf* ^*Enh170*^ to sequences upstream of the *Bdnf* gene (Figure 2B).

To verify the loop of *Bdnf* to the distal interacting site and assess its frequency more quantitatively, DNA-FISH was performed using fosmids encompassing a) the *Bdnf* ^*Enh170*^ and *Lin7c* gene, b) the *Bdnf* gene, and c) a control downstream region located the same distance from *Bdnf* ^*Enh170*^ as *Bdnf* (170 kb). Measuring the distance between these probes in pairwise combinations revealed that in PMNs, *Bdnf* ^*Enh170*^ was closer to, and exhibited more frequent interactions with, the *Bdnf* probe compared to the downstream probe (Figure 2C). Importantly, *Bdnf* ^*Enh170*^ and the *Bdnf* gene regions were in closer proximity in PMNs than in NPCs, and co-localization frequency increased during differentiation (Figure 2C). Thus, although the looping profiles are similar at the population level (Figure 2A), single cell analysis indicated more frequent interactions between *Bdnf* ^*Enh170*^ and *Bdnf* taking place in cells where *Bdnf* expression is high (Figure 2C). The use of a reciprocal combination of probe labels further supported this conclusion (Figure S2). Our findings are in accordance with previous studies showing that chromosome conformation capture technologies usually capture *proximity* of enhancers and promoters, whereas DNA-FISH can detect *direct interactions* between genomic regions.^49^^,^^50^

### *Bdnf*^*Enh170*^ bears many characteristics typical of enhancers

**Figure 3:**
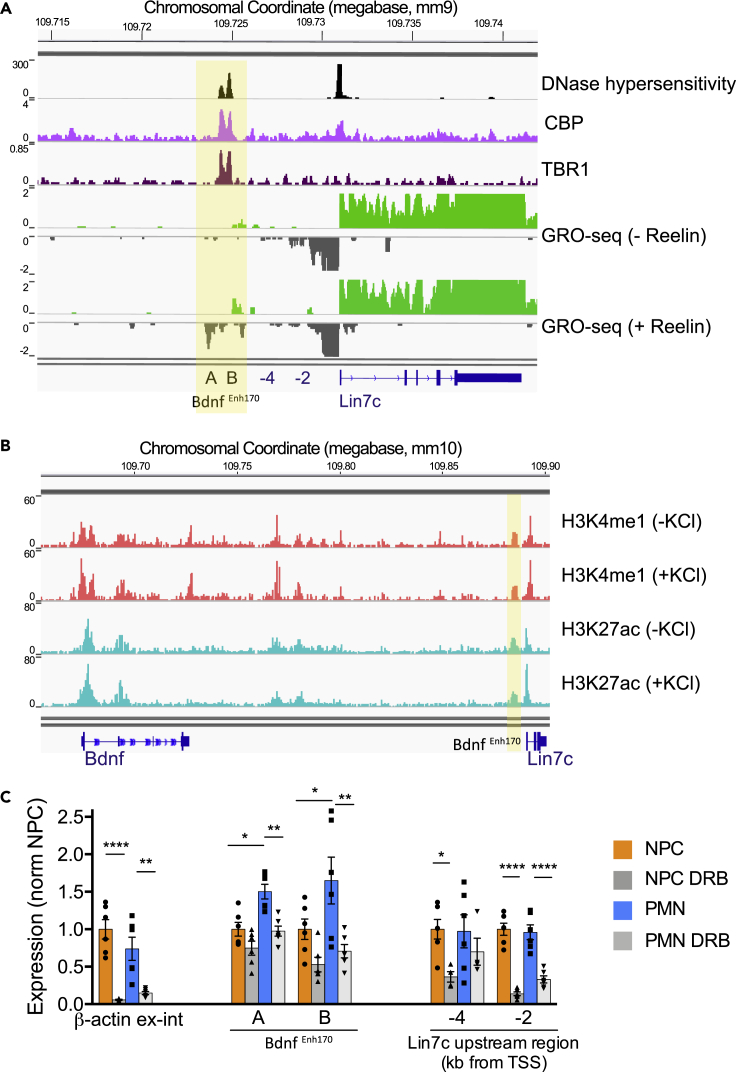
The Bdnf-interacting intergenic region displays hallmarks of an enhancer (A) Available data for DNaseI hypersensitivity, and CBP (CREB Binding Protein) and TBR1 (T-Box Brain Transcription Factor 1) ChIP-seq were visualized at *Bdnf*^*Enh170*^ in brain or neurons. GRO (Genome Run On)-seq profiles from control or Reelin-treated cortical neurons show nascent transcription at *Bdnf*^*Enh170*^ in stimulated cortical neurons. A and B mark sites positive for GRO-seq signal within the putative enhancer region used for qRT-PCR verification. −4 and −2 indicate location of primers used to assess transcription in *Lin7c* upstream region in qRT-PCR. (B) H3K4me1 and H3K27ac ChIP-seq in cortical neurons minus and plus KCl show enhancer hallmarks at *Bdnf*^*Enh170*^. (C) qRT-PCR expression analysis of two regions within *Bdnf*^*Enh170*^ in untreated or DRB-treated (5,6-dichloro-1-β-D-ribofuranosylbenzimidazole RNA polymerase II inhibitor) NPC and PMN. Data normalized to NPC. *β-actin* primary transcripts (primers amplifying exon-intron junction) shown to demonstrate DRB treatment efficacy. Regions between *Bdnf*^*Enh170*^ and *Lin7c* shown to demonstrate that enhancer is a separate transcriptional unit. Bars represent mean ± SEM, and points show results from different biological replicates (n = 6). ∗p< 0.05, ∗∗p< 0.01, ∗∗∗p< 0.001, ∗∗∗∗p< 0.0001, two-way ANOVA with Sidak’s multiple comparisons test. β-actin ex-int: p< 0.0001 (NPC Unt versus DRB), p = 0.0011 (PMN Unt versus DRB). Enh-A: p = 0.0009 (PMN Unt versus DRB), p = 0.0014 (Unt NPC versus PMN). Enh-B: p = 0.0032 (PMN Unt versus DRB), p = 0.0410 (Unt NPC versus PMN). Lin7c -4kb: p = 0.0436 (NPC Unt versus DRB). Lin7c -2kb: p< 0.0001 (NPC Unt versus DRB), p< 0.0001 (PMN Unt versus DRB). See also Figure S3 and Table S1.

To assess whether *Bdnf* ^*Enh170*^ exhibits the characteristics of an enhancer, we first analyzed publicly available data. Sensitivity to DNase I is a feature of active chromatin regions including promoters and enhancers.^51^ ENCODE DNase I hypersensitivity data showed peaks at *Bdnf* ^*Enh170*^ in brain (Figure 3A), but not in other tissues, which was similar to the pattern of DNase I hypersensitivity observed at *Bdnf* promoters (Figure S3A). A dataset using an alternative chromatin accessibility assay named Assay for Transposase-Accessible Chromatin with Sequencing (ATAC-seq^52^), also identified open chromatin at *Bdnf* ^*Enh170*^ in microdissected hippocampal dentate gyri (not shown).

We then investigated other enhancer hallmarks at *Bdnf* ^*Enh170*^ using ChIP-seq datasets generated by our and other laboratories (Table S1^53^^,^^54^^,^^55^). Chromatin modifications, such as H3K4me1 and H3K27ac, predict enhancer function genome-wide.^56^^,^^57^,^58^ The histone acetyltransferase CBP (CREB Binding Protein) is an enhancer regulator which catalyses the addition of H3K27ac.^59^^,^^58^ The transcriptional coactivator Mediator interacts with cohesin to regulate enhancer-promoter looping.^60^ Enhancer sites recruit multiple transcription factors.^28^ We identified a strong peak of the enhancer epigenetic signatures H3K27ac and H3K4me1 at *Bdnf* ^*Enh170*^ in basal and depolarized cortical neurons (Figure 3B). CBP and the MED23 Mediator subunit were also found to bind to *Bdnf* ^*Enh170*^ in cortical neurons (Figures 3A and S3B), together with the transcription factors MEF2, CREB and TBR1 (Figures 3A and S3B). The transcription factors and coactivators show a double peak at *Bdnf* ^*Enh170*^, coinciding with a double peak of DNase I hypersensitive sites.

Enhancers are transcribed in many cell types, including neurons.^53^^,^^54^^,^^61^ In some instances, the enhancer RNA (eRNA) has functional roles, such as interacting with Negative Elongation Factor,^62^ CBP,^63^ or RNA Polymerase II (RNAPII^53^), or affecting 3D contacts.^64^ In other systems, eRNA transcription may contribute to the maintenance of the transcriptional machinery or the opening of the chromatin.^65^^,^^66^ Regardless of mechanism, the production of eRNAs is considered a critical feature of active enhancers. We therefore sought to determine whether transcriptional activity could be detected from *Bdnf* ^*Enh170*^. eRNAs are lowly expressed and unstable, and conventional RNA-seq databases may not show transcription at enhancer sites. Methods that detect nascent RNA such as Genome Run On with sequencing (GRO-seq) are better suited for detecting eRNAs, because they map transcripts actively engaged with RNAPII.^67^ Analysis of GRO-seq data from Reelin-stimulated cortical neurons,^54^ showed that RNA is transcribed bidirectionally from the *Bdnf* ^*Enh170*^ region (Figure 3A). As expected, the *Lin7c* gene exhibited bidirectional RNA production at the active promoter.^67^^,^^68^

To validate the sequencing data, qRT-PCR was performed on NPCs and PMNs using primers that generate amplicons within the region of GRO-seq enrichment (Figure 3A, sites A and B). Because eRNA are transcribed at very low levels, cells were treated with the transcriptional inhibitor DRB (5,6-dichloro-1-beta-D-ribofuranosylbenzimidazole) to determine background levels. We found that *Bdnf* ^*Enh170*^ was transcribed in PMNs, at levels significantly higher than either in NPCs or in DRB-treated PMNs (Figure 3C). A region just downstream of the enhancer (−4.0 kb from the *Lin7c* transcriptional start site) showed no increase in transcription from NPC to PMN, and no sensitivity to DRB in PMN (Figure 3C), confirming that the eRNAs are not a continuation of *Lin7c* promoter antisense transcripts.

Together, these findings demonstrate that an intergenic region interacting with *Bdnf* in neurons possesses most enhancer hallmarks, and is transcribed in PMNs when *Bdnf* gene expression is high.

### *Bdnf*^*Enh170*^ regulates *Bdnf* expression during neuronal differentiation

**Figure 4:**
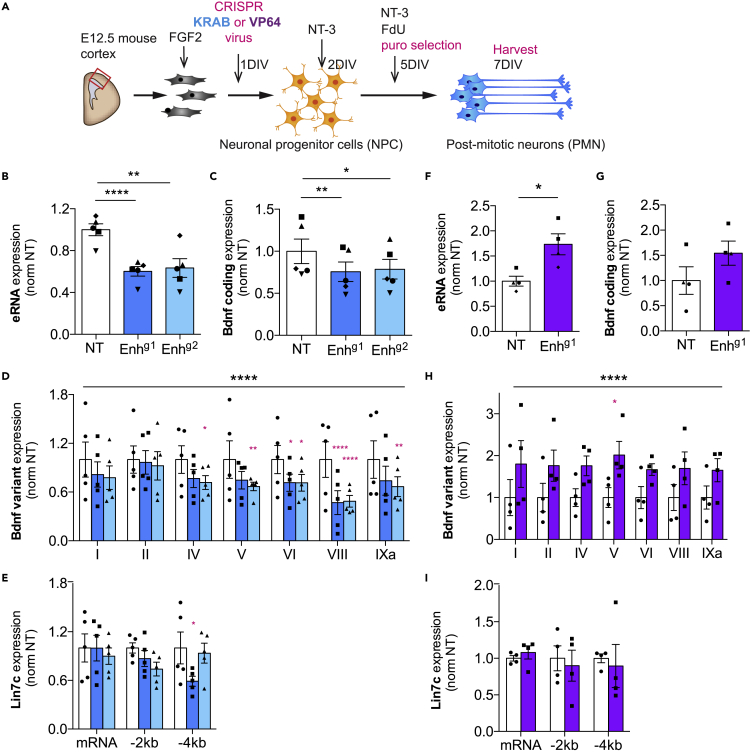
*Bdnf*^*Enh170*^ is a positive regulator of *Bdnf* expression during differentiation (A) Schematic of CRISPR virus experiments. (B–E) qRT-PCR of PMN targeted with lentiviral dCas9-KRAB targeted by no guide (NT, nontargeting; open bars) or guides against *Bdnf*^*Enh170*^ (Enh^g1^, Enh^g2^; blue bars). Data are normalized to NT-transduced cells. Bars represent mean ± SEM, and points show different biological replicates (n = 5). ∗p< 0.05, ∗∗p< 0.01, ∗∗∗p< 0.001. (B) Expression profile of *Bdnf*^*Enh170*^ enhancer RNA (eRNA). Paired one-way ANOVA: *F* = 42.93, p = 0.0027. Dunnett’s multiple comparisons: Empty versus Enh^g1^ p< 0.0001; Empty versus Enh^g2^ p = 0.0023. (C) Expression profile of *Bdnf* coding mRNA. Paired one-way ANOVA: *F* = 15.93, p = 0.0067. Dunnett’s multiple comparisons: Empty versus Enh^g1^ p = 0.0063; Empty versus Enh^g2^ p = 0.0417. (D) Expression profile of *Bdnf* variants. Two-way ANOVA with Sidak’s multiple comparison test (black asterisks, overall p value; red asterisks, multiple comparison p value; see STAR Methods). (E) Expression profile of *Lin7c* variants. Two-way ANOVA with Sidak’s multiple comparison test (see STAR Methods). (F–I) qRT-PCR of PMN targeted with lentiviral dCas9-VP64 targeted by no guide (NT, open bars) or a guide against the enhancer (Enh^g1^; purple bars). Data are normalized to NT-transduced cells. Bars represent mean ± SEM, and points show different biological replicates (n = 4). ∗p< 0.05, ∗∗p< 0.01, ∗∗∗p< 0.001. (F) Expression profile of *Bdnf*^*Enh170*^ enhancer RNA (eRNA). Paired t-test (two-tailed): p = 0.0296, *t* = 3.915, *df* = 3. (G) Expression profile of *Bdnf* coding mRNA. Paired t-test (two-tailed): p = 0.1043, *t* = 2.307, *df* = 3. (H) Expression profile of *Bdnf* variants. Two-way ANOVA with Sidak’s multiple comparison test (black asterisks, overall p value; red asterisks, multiple comparison p value; see STAR Methods). (I) Expression profile of *Lin7c* variants. Two-way ANOVA with Sidak’s multiple comparison test (see STAR Methods).

To test whether *Bdnf* ^*Enh170*^ regulates *Bdnf* expression during NPC differentiation we employed RNA-guided Clustered Regularly Interspaced Palindromic Repeats inhibition (CRISPRi). A catalytic mutant Cas9 (dCas9) was fused to the transcriptional inhibitor KRAB (Krüppel-associated box^69^), and lentivirus was generated either in combination with no targeting (NT) guide RNA (gRNA), or a gRNA targeted to *Bdnf* ^*Enh170*^ (Enh^g1^ or Enh^g2^). NPCs were infected with CRISPRi lentivirus expressing a puromycin resistance cassette and allowed to differentiate *in vitro*; neurons were selected for two days before harvesting PMNs (Figure 4A). Expression of *Bdnf* ^*Enh170*^ eRNA after differentiation was significantly decreased in the presence of enhancer-targeted gRNAs (Figure 4B). *Bdnf* ^*Enh170*^ inhibition caused a significant reduction of *Bdnf* total mRNA (measured in the universal exon) confirming that that it is a functional *Bdnf* enhancer (Figure 4C). Analysis of different *Bdnf* isoforms indicated that *Bdnf* ^*Enh170*^ inhibition resulted in lower expression of most *Bdnf* variants, with significant effects on isoforms expressing exon IV, V, VI, VIII or IXa (Figure 4D). We did not see a reduction in *Lin7c* mRNA or antisense promoter transcription at −2.0 kb (Figure 4E), confirming that the CRISRPi inhibitory effect at the enhancer does not spread into the adjacent *Lin7c* promoter.

To further investigate the link between enhancer and variant transcription, we performed these experiments using dCas9 fused to the transcriptional activator VP64.^70^ The targeting of CRISPRa (activator) virus complexes to *Bdnf* ^*Enh170*^ using the same Enh^g1^ increased eRNA transcription (Figure 4F). *Bdnf* total mRNA showed an increase after *Bdnf* ^*Enh170*^ activation, albeit not statistically significant (Figure 4G)*. Bdnf* variants as a group showed increased expression, with significance seen for exon V (Figure 4H). No changes were observed for *Lin7c* mRNA or antisense transcription (Figure 4I). These experiments confirm that *Bdnf* ^*Enh170*^ is a *bona fide* enhancer of developmental *Bdnf* expression.

### *Bdnf*^*Enh170*^ regulates activity-dependent *Bdnf* expression in cortical neurons

*Bdnf* gene expression is increased after neuronal stimulation as well as during differentiation. To investigate whether *Bdnf* ^*Enh170*^ contributed to activity-dependent *Bdnf* induction, we first investigated whether *Bdnf* ^*Enh170*^ is transcribed in response to neuronal activation. Primary cortical neurons were depolarized with KCl, and eRNA levels were assessed with qRT-PCR. *Bdnf* ^*Enh170*^ eRNA increased concomitant with *Bdnf* mRNA (Figure S4A). The activity-dependent Activator Protein-1 (AP-1) transcription factors JUN and FOS were recruited to *Bdnf* ^*Enh170*^ in response to neuronal depolarization (Figure S4B), which is consistent with transcription factors encoded by early response genes, like *Fos* and *Jun*, controlling the expression of late response genes, such as *Bdnf*.^71^

To assess whether the enhancer was required for activity-dependent *Bdnf* induction, CRISPRi (dCas9-KRAB) lentivirus was generated either in combination with no targeting gRNA (NT) or targeted to the putative enhancer region (Enh^g1^, Enh^g2^), as before. CRISPRi lentivirus was added to primary cortical neurons on the same day as dissociation, and the media was changed the following day. After 5 DIV, the media was supplemented with puromycin to select lentiviral-transduced cells, and then cells were depolarized with KCl at DIV 7 for 3h before harvesting (Figure S4C). Expression of *Bdnf* ^*Enh170*^ eRNA after neuronal activation was significantly decreased in the presence of enhancer-targeted guide RNAs (Figure S4D). Although we did not detect a significant reduction of total *Bdnf* mRNA after *Bdnf* ^*Enh170*^ inhibition (Figure S4E), analysis of different *Bdnf* isoforms indicated that *Bdnf* ^*Enh170*^ inhibition resulted in lower expression of *Bdnf* variants as a group (Figure S4F), confirming its role as an enhancer. Significant effects of *Bdnf* ^*Enh170*^ inhibition were seen on variants containing exon II and V (Figure S4F). We did not see a reduction in *Lin7c* mRNA or antisense promoter transcription at −2.0 kb (Figure S4G), confirming that the CRISRPi inhibitory effect at the enhancer does not spread into the adjacent *Lin7c* promoter. The effects of enhancer inhibition on *Bdnf* expression were more subtle after depolarization than during differentiation, and the variants primarily affected were different. This is consistent with the hypothesis that *Bdnf* gene expression may depend on several enhancers, which are activated in a combinatorial manner depending on physiological context.

### *Bdnf*^*Enh170*^ regulates Bdnf-dependent dendritogenesis in cortical neurons

**Figure 5:**
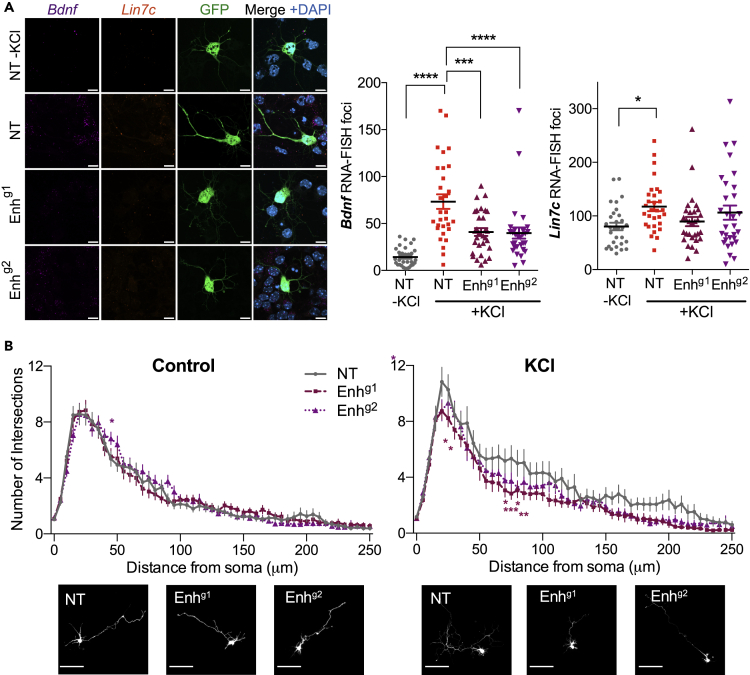
*Bdnf*^*Enh170*^ is required for activity-dependent *Bdnf* expression and neuritic complexity Cortical neurons were transfected with a GFP expression vector (pBIRD) in combination with dCas9-KRAB-MECP2 and an expression vector for guide RNAs (Non-targeting (NT) or targeting *Bdnf*^*Enh170*^ (Enh^g1^ or Enh^g2^)). Cells were maintained under basal or depolarizing (50 mM KCl, 48h) conditions. (A) Quantitative HCR (hybridization chain reaction) RNA-FISH demonstrated that CRISPR inhibition of *Bdnf*^*Enh170*^ reduces *Bdnf* (magenta puncta), but not *Lin7c* (orange puncta), expression following depolarization in transfected (GFP-positive) cortical neurons. Left panel, representative images. Scale bar, 10 μm. Right, quantitation. Line and error bars, mean ± SEM Each point represents a cell, n = 30 across 3 biological replicates. ∗p< 0.05, ∗∗p< 0.01, ∗∗∗p< 0.001, ∗∗∗∗p< 0.0001. *Bdnf* one-way ANOVA (*F* = 20.19, p< 0.0001) with Dunnett’s multiple comparisons test: NT versus NT-KCl p< 0.0001; NT versus Enh^g1^, p = 0.0001; NT versus Enh^g2^p< 0.0001. *Lin7c* one-way ANOVA (*F* = 2.942, p = 0.0360) with Dunnett’s multiple comparisons test: NT versus NT-KCl p = 0.0213. (B) GFP immunostaining and dendritic tracing of transfected neurons shows a lack of dendritic branching in neurons after enhancer inhibition. Top, Sholl analysis of the dendritic processes of 30 neurons per treatment (10 per biological replicate). For each distance point, the mean ± SEM is shown. ∗p< 0.05, ∗∗p< 0.01, ∗∗∗p< 0.001, ∗∗∗∗p< 0.0001. Control two-way ANOVA (p = 0.0113) with Sidak’s multiple comparisons test: NT versus Enh^g2^ p = 0.0053 (45 μm). KCl two-way ANOVA (p< 0.0001) with Sidak’s multiple comparisons test: NT versus Enh^g1^ p = 0.0120 (20 μm), p = 0.0211 (25 μm), p = 0.0120 (70 μm), p = 0.0003 (75 μm), p = 0.0475 (80 μm), p = 0.0067 (85 μm); NT versus Enh^g2^ p = 0.0211 (20 μm). For full details see STAR Methods. Lower, representative images. Scale bar, 100 μm. See also Figures S4 and S5.

We next sought to study whether *Bdnf* ^*Enh170*^ promoted the physiological functions of Bdnf in cortical neurons after stimulation. *Bdnf* expression is necessary for activity-dependent dendritogenesis,^72^^,^^73^ a critical process for neuronal growth at later stages of development. To study how *Bdnf* ^*Enh170*^ promotes activity-dependent dendritogenesis, cortical neurons were transfected with plasmids encoding dCas9-KRAB-MECP2,^74^ with a plasmid encoding the same gRNAs used to knockdown expression in the lentiviral system (BPK1520 vector: NT, Enh^g1^, Enh^g2^) and a GFP-encoding plasmid. dCas9-KRAB-MECP2 is a potent repressor in neurons,^75^ and because of the single cell nature of the assays it was important to ensure that a strong inhibition was taking place at individual loci. Neurons were maintained either in basal or depolarizing conditions (50 mM KCl, 48 h), and GFP-positive, non-overlapping neurons were analyzed. Quantitative hybridization chain reaction (HCR) RNA-FISH with *Bdnf* and *Lin7c* probes confirmed that in NT cortical neurons, we could detect an increase in *Bdnf* and *Lin7c* mRNA after depolarization (Figure 5A), as expected. Inclusion of a gRNA targeting *Bdnf* ^*Enh170*^ decreased *Bdnf* but not *Lin7c* total mRNA levels in stimulated neurons (Figure 5A), further confirming that *Bdnf* ^*Enh170*^ enhances activity-dependent *Bdnf* expression.

Dendritic tracing and Sholl analysis showed that, as expected, depolarization induced a significant increase in dendritic complexity in control neurons transfected with dCas9-KRAB-MECP2 only (NT, Figure 5B). KCl-dependent dendritic branching was substantially reduced when neurons were transfected with dCas9-KRAB-MECP2 targeted to *Bdnf* ^*Enh170*^ (Enh^g1^ or Enh^g2^), with no effect on basal arborization (Figure 5B). To assess whether the effect of *Bdnf* ^*Enh170*^ inhibition was rescued by Bdnf, dendritogenesis was assessed in neurons expressing a vector encoding either the *Bdnf* coding sequence (pBdnf) or an empty control vector (EV), and co-transfected with CRISPRi vectors (NT or Enh^g2^). Depolarization of cortical neurons in the presence of EV increased neuritic arborization, which was reduced by *Bdnf* ^*Enh170*^ inhibition (Figure S5). Co-transfection of pBdnf rescued the branching defects close to the soma, although it did not fully reinstate the branching in distal dendrites (Figure S5). Together these findings indicate that *Bdnf* ^*Enh170*^ regulates *Bdnf* expression to promote dendritogenesis.

### *Bdnf*^*Enh170*^ influences neuronal differentiation and cortical development *in vivo*

Bdnf and its main receptor TrkB play a critical role in mouse cortical development, chiefly by regulating neuronal progenitor proliferation^76^ and neural migration.^76^^,^^77^ We next investigated whether *Bdnf* ^*Enh170*^ may promote these developmental functions of Bdnf. Initial experiments performed on NPCs in culture indicated that inhibition of *Bdnf* ^*Enh170*^ affected PMN cluster formation, quantified by measuring nuclei-nuclei distance (Figures S6A and S6B). PMN dispersion was reversed by co-infection with a lentiviral vector encoding *Bdnf* (Figure S6C), indicating that Bdnf is necessary for neuron-neuron interaction, cell migration, or adhesion properties *in vitro*. The expression of markers of neuronal differentiation such as Map2, NeuN, and Nestin was unchanged (not shown).

**Figure 6:**
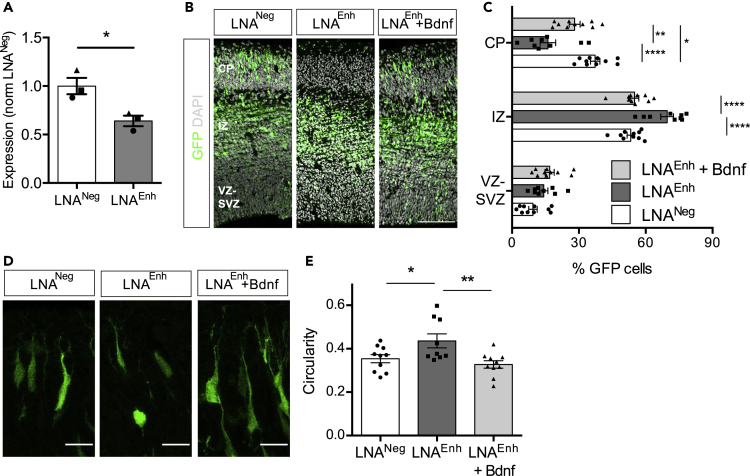
*Bdnf*^*Enh170*^ is required for cortical development (A) *Bdnf*^*Enh170*^ enhancer RNA expression decreases following LNA^Enh^ treatment of PMNs. Levels of *Bdnf*^*Enh170*^ eRNA in PMN transfected with LNA^Neg^ or LNA^Enh^ for 48h before harvest, assessed by qRT-PCR and normalized to LNA^Neg^ samples. Bars represent mean ± SEM, and points show values of different biological replicates (n = 4). ∗p< 0.05, paired t-test (two-tailed). LNA^Neg^ versus LNA^Enh^ p = 0.0155, *t* = 7.940, *df* = 2. (B) E13.5 embryonic brains were electroporated *in utero* with LNAs and a CAG-Bdnf-expressing construct as indicated, and then analyzed at E15.5. Shown are representative images of coronal sections immunolabeled for GFP (green) and DAPI (gray). Scale bar, 100 μm. (C) Quantification of the distribution of cells electroporated as in B between the ventricular-subventricular (VZ-SVZ), intermediate zone (IZ), and cortical plate (CP). Data are from 9 to 10 embryos per condition across 3–4 independent experiments. Bars represent mean ± SEM, and points show values of different embryos. ∗p< 0.05, ∗∗p< 0.01, ∗∗∗p< 0.001, ∗∗∗∗p< 0.0001, two-way ANOVA with Tukey’s post test. CP: LNA^Neg^ versus LNA^Enh^p< 0.0001; LNA^Neg^ versus LNA^Enh^ + Bdnf p = 0.0127; LNA^Enh^ versus LNA^Enh^ + Bdnf p = 0.0012. IZ: LNA^Neg^ versus LNA^Enh^p< 0.0001; LNA^Enh^ versus LNA^Enh^ + Bdnf p< 0.0001. For full details see STAR Methods. (D) E13.5 embryonic brains were electroporated *in utero* as in B and then the circularity of neurons in the cortical plate was analyzed. Shown are representative images of coronal sections immunolabeled for GFP (green). Scale bar, 15 μm. (E) Quantitation of the circularity of cortical plate cells. Data analyzed from the same embryos as B (9–10 embryos per condition across 3–4 independent experiments). Bars represent mean ± SEM, and points show values of different embryos (average of multiple cells per embryo). ∗p< 0.05, ∗∗p< 0.01, one-way ANOVA (p = 0.0080, *F* = 5.847), with Tukey’s post test: LNA^Neg^ versus LNA^Enh^ p = 0.0491; LNA^Enh^ versus LNA^Enh^ + Bdnf p = 0.0076. See also Figure S6.

Finally, we investigated whether *Bdnf* ^*Enh170*^ could affect cortical development *in vivo*. The mouse cortex is formed in a characteristic inside-out manner, with deep layers generated first and more superficial layers generated later.^78^ Neurons are generated in the ventricular zone (VZ) and initially populate the deeper layers of the cortex, whereas neurons born at later developmental stages must cross the deeper layers of the cortex to form the upper layers. To ask whether *Bdnf* ^*Enh170*^ affected neuronal cell migration, we employed *in utero* intracerebroventricular injection with electroporation. Locked Nucleic Acids (LNAs, Qiagen) were used to specifically target *Bdnf* ^*Enh170*^ eRNA for degradation, because of the toxicity of large CRISPRi plasmids *in vivo*. We identified an LNA that significantly reduced the levels of *Bdnf* ^*Enh170*^ eRNA in PMNs (LNA^Enh^; Figure 6A). Control LNA^Neg^ or LNA^Enh^ were electroporated together with a GFP expression plasmid into E13.5 mouse brains and, after 2 days of *in vivo* development, migration of GFP-positive neurons to the cortical plate (CP) was assessed. After 48h, 37% of the neuronal progenitors electroporated with LNA^Neg^ in VZ had exited the cell cycle and migrated to reach the CP (Figures 6B and 6C), in keeping with previous observations.^38^^,^^53^ Reduction of *Bdnf* ^*Enh170*^ eRNA expression resulted in an accumulation of cells within the intermediate zone (IZ), and a significant reduction in neurons that migrated into the CP (Figures 6B and 6C). Co-electroporation of neuronal progenitors with a vector expressing the *Bdnf* coding region under the control of a cytomegalovirus (CMV) early enhancer/chicken β-actin (CAG) promoter partially rescued the defects induced by *Bdnf* ^*Enh170*^ eRNA inhibition (Figures 6B and 6C). Taken together, these data demonstrate that *Bdnf* ^*Enh170*^ is necessary for cortical development and that knockdown of its eRNA results in neuronal migration defects.

During the development of the cortex, cortical neurons use multipolar migration to move from their birthplace, and then establish polarity to enable bipolar radial migration.^79^ Once they reach their position in the cortex, they develop axons and dendrites and form connections.^79^ Because of the interlinked nature of radial migration and neurite outgrowth,^78^ and the importance of Bdnf for dendritic tree elaboration^72^^,^^80^^,^^81^^,^^82^ the circularity of the neurons that reach the cortical plate was analyzed (Figures 6D and 6E). Knockdown of *Bdnf* ^*Enh1*^ eRNA using LNA^Enh^ increased the circularity of neurons within the cortical plate (Figures 6D and 6E), suggesting that *Bdnf* ^*Enh170*^ influenced neuron shape and neurite branching. This effect was rescued by expression of the *Bdnf* coding region (Figures 6D and 6E).

## Discussion

The *Bdnf* gene has a complex genomic structure . In mice, it comprises at least nine 5′ untranslated exons, each containing a promoter that is alternatively spliced to a common translated coding exon. Despite intensive scrutiny, the role of most promoters in regulating *Bdnf* expression remains unclear. Here, we identify *Bdnf* ^*Enh170*^ as a novel intergenic enhancer that influences *Bdnf* expression during cortical development and in response to neuronal depolarization. *Bdnf* ^*Enh170*^ can increase the expression of many *Bdnf* isoforms during neuronal differentiation (Figures 4F–4I). Its inhibition significantly regulates the expression of at least five *Bdnf* 5′ isoforms during differentiation (Figure 4D), and at least two in response to depolarization (Figure S4F). *Bdnf* ^*Enh170*^ bears most known enhancer hallmarks, including binding of CBP and Mediator, chromatin accessibility, specific histone modifications, and transcription (Figures 3 and S3). Analysis of genome topology revealed that *Bdnf* gene and enhancer are located within a sub-TAD which is bounded by CTCF and cohesin (Figures S1B and S1C). *Bdnf* activation correlates with increasing frequency of enhancer-promoter co-localization (Figures 2C and S2) and movement of the genomic region away from the nuclear periphery (Figure 1D). *Bdnf* ^*Enh170*^ inhibition altered neuronal clustering (Figure S6), dendritic branching (Figures 5B and S5), and cortical development (Figure 6), demonstrating its key role in regulating Bdnf functions *in vitro* and *in vivo*.

We explored the 3D genome architecture of the *Bdnf* genomic region using HiC and 4C-seq and described, for the first time, a sub-TAD of increased interaction frequency that includes the *Bdnf* gene and the downstream region including *Bdnf* ^*Enh170*^ and the neuronal gene *Lin7c*, with CTCF and cohesin-positive boundaries within the *Bdnf* and *Lin7c* genes (Figures 2A, 2B, S1B, and S1C). Neither the sub-TAD boundaries nor the enhancer-promoter loop sites changed during neuronal differentiation (Figures 2A and S1B), suggesting that the boundaries are pre-wired in NPCs. Topological structure has been shown to precede gene activation in many model systems,^83^^,^^84^^,^^85^^,^^86^^,^^87^ and preconfigured loops prime genes for transcriptional activation.^88^ Single cell imaging showed an increase in co-localization of *Bdnf* ^*Enh170*^ with the *Bdnf* promoter during the activation of the gene (Figures 2C and S2), suggesting that the sub-TAD organization may facilitate enhancer-promoter interaction and transcription.

Importantly, the enhancer-promoter loop that we identified with 4C-seq was also found in a recent study that examined the topology of the *Bdnf* genomic region using 5C technology in cortical neurons.^36^ In addition to constitutive loops, the authors also described loops which form in response to depolarization.^36^ Future investigations will clarify the functional significance of these loops on *Bdnf* expression, and the interplay with the intergenic enhancer characterized in our study.

To explore the promoter selectivity of *Bdnf* ^*Enh170*^ we performed gain and loss of function experiments. CRISPRa experiments (Figures 4F–4I) demonstrated that all *Bdnf* variants can be regulated by *Bdnf* ^*Enh170*^. However, different *Bdnf* variants were downregulated to different extents by CRISPRi, which may be influenced by the expression level and stability of each isoform, as well as the physiological context. During differentiation, *Bdnf* ^*Enh170*^ inhibition markedly affected isoforms containing exon IV, V, VI, VIII, and IXa (Figure 4), whereas in response to depolarization, the strongest effect of *Bdnf* ^*Enh170*^ inhibition was on exon V and exon II- expressing isoforms (Figure S4F). These findings demonstrate that *Bdnf* regulation is dependent on physiological context and suggests the existence of additional enhancers or modulators of enhancer activation.

A recently described *Bdnf* intronic enhancer^37^ has been shown to promote transcription only from *Bdnf* promoters I, II, and III in response to neuronal activation.^37^ In addition to different promoter-selectivity, *Bdnf* ^*Enh170*^ inhibition decreased total *Bdnf* mRNA expression during differentiation of cultured neurons (Figure 4C), which is in contrast to the effect observed on the inhibition of the intronic enhancer,^37^further confirming their distinct role in regulating *Bdnf* expression.

We found that *Bdnf* ^*Enh170*^ inhibition has significant physiological consequences for neuronal differentiation and development both *in vitro* and *in vivo*. In addition to promoting the formation of neuronal clusters (Figure S6) and dendritogenesis (Figures 5 and S5), *Bdnf* ^*Enh170*^ is necessary for mouse cortical neuron development *in vivo* (Figure 6). Inhibition of *Bdnf* ^*Enh170*^ expression disrupts neuronal migration, which was restored by co-electroporation with a vector expressing Bdnf (Figures 6B and 6C). The effect on neuronal migration could be linked to the neurite extension phenotype seen *in vitro* (Figures 5 and S5) because neuronal polarity and morphology are tightly interlinked with migration.^78^ Moreover, *Bdnf* ^*Enh170*^ inhibition caused a loss of bipolar morphology in neurons that reached the cortical plate, which was restored by co-electroporation with a vector expressing Bdnf (Figures 6D and 6E).

Inhibition of the Bdnf receptor TrkB has been shown to reduce neuronal progenitor proliferation in the VZ and neuronal migration.^76^^,^^77^ Our results however indicate that *Bdnf* ^*Enh170*^ affects principally the expression of *Bdnf* during neuronal radial migration, with little or no effect on cell proliferation. A possible explanation is that neuronal progenitor proliferation may depend on Bdnf encoded by mRNA variants that are not regulated by *Bdnf* ^*Enh170*^. Investigation of the importance of the intronic enhancer^37^ and other putative *Bdnf* enhancers^36^*in vivo* will elucidate the complex regulation of Bdnf during cortical development.

Transcriptional regulation of the *BDNF* gene has important implications for the pathogenesis of many neurological disorders. *Bdnf* ^*Enh170*^ was identified in mouse but is conserved in the human genome, where it is in a similar orientation and position relative to the *BDNF* and *LIN7C* genes (not shown). In humans, an antisense transcript that regulates *BDNF* expression, *BDNF-AS*, runs from immediately upstream of the *LIN7C* transcriptional start site through the entire intergenic region and the *BDNF* gene.^18^^,^^89^ Further investigation will address whether, as for the mouse gene, multiple enhancers regulate the human *BDNF* gene, determining distinct spatiotemporal expression patterns that may be perturbed in neurological disorders.

### Limitations of study

Most experiments for this study were performed using *ex vivo* cultured and differentiated cortical neurons, which may compromise the relevance to *in vivo* differentiation processes. The CRISPRi studies were done with two independent guide RNAs to reduce enhancer RNA expression, however off-target effects cannot be ruled out. Although the efficiency of the LNA GapmeRs at reducing enhancer RNA expression was demonstrated, the effect on *Bdnf* mRNA expression could not be verified because of low transfection, and was not verified *in vivo* where the LNAs were used. As for all assays based on microscopy, quantification of fluorescence based on images may be less accurate than other methods.

## STAR★Methods

### Key resources table

**Table:** 

REAGENT or RESOURCE	SOURCE	IDENTIFIER
**Antibodies**		

Sheep anti-digoxigenin fluorescein fab fragments	Roche	Cat#: 11207741910; RRID:AB_514498
Rabbit anti-sheep antibodies fluorescein	Vector Labs	Cat#: FI-6000,; RRID:AB_2336218
Streptavidin-555	Thermo Fisher Scientific	Cat#: S32355; RRID:AB_2571525
Rabbit anti-Rad21 antibody	Abcam	Cat#: ab992; RRID:AB_2176601
Chicken anti-GFP antibody	Abcam	Cat#: ab13970; RRID:AB_300798, 1:2000
Mouse anti-mCherry	Abcam	Cat#: ab125096; RRID:AB_11133266
Goat anti-chicken AlexaFluor-488	Thermo Fisher Scientific	Cat#: A-11039; RRID:AB_2534096
Donkey anti-mouse AlexaFluor-555	Thermo Fisher Scientific	Cat#: A-31570; RRID:AB_2536180

**Chemicals, peptides, and recombinant proteins**		

Recombinant human Neurotrophin-3 (NT-3) protein	Alomone	N-260
Recombinant human FGF basic (FGF-2/bFGF) (aa 1-155) protein	Thermo Fisher Scientific	PHG0264
Tetrodotoxin citrate	Abcam	ab120055
DpnII	NEB	R0543L
Csp6I	Thermo Fisher Scientific	ER0211
5,6-dichloro-1-β-D-ribofuranosylbenzimidazole (DRB) RNA polymerase II inhibitor	Merck	D1916
ProLong Gold antifade mountant	Thermo Fisher Scientific	P36930
PEImax	Polysciences	24765

**Critical commercial assays**		

Nick Translation Kit	Roche	10976776001
Expand Long Template polymerase	Roche	1168184201
Qubit high sensitivity assay	Thermo Fisher Scientific	Q32851
MiSeq Reagent Kit v3, 150-cycle	Illumina	MS-102-3,001

**Deposited data**		

4C-seq	This paper	GEO: GSE190306

**Experimental models: organisms/strains**		

Primary neuronal progenitor cells isolated from E12.5 C57BL6 mouse cortex and differentiated to postmitotic neurons	This paper	NA
Primary cortical neurons isolated from E15.5 C57BL6 mouse cortex	This paper	NA
C57BL/6J mice	The Jackson Laboratory	Strain #:000664; RRID:IMSR_JAX:000664

**Oligonucleotides**		

Guide RNA 1 targeting *Bdnf*^*Enh170*^ (Enh^g1^) GGATTGTTTGGACTTACTCT	This paper	NA
Guide RNA 2 targeting *Bdnf*^*Enh170*^ (Enh^g2^) GGATTGTTTGGACTTACTCT	This paper	NA
Primers for 4C-seq; see Table S2	This paper	NA
Primers for qPCR, ChIP; see Table S3	This paper	NA

**Recombinant DNA**		

Plasmid BPK1520	Kleinstiver et al.^90^	Addgene 65777
Plasmid dCas9-KRAB-MECP2	Yeo et al.^74^	Addgene 110821
Plasmid pLV hU6-sgRNA hUbC-dCas9-KRAB-T2a-puro	Thakore et al.^69^	Addgene 71236
Plasmid pLV hU6-sgRNA hUbC-dCas9-KRAB-T2a-GFP	Thakore et al.^69^	Addgene 71237
Plasmid pLV hU6-sgRNA hUbC-dCas9-VP64-T2a-puro	See CRISPR-Cas9 section of STAR methods of this paper	NA
Plasmid NLS-RFP-P2A-SypGFP	Sampathkumar et al.^91^	NA
Plasmid NLS-RFP-P2A-SypGFP-T2A-BDNF	Sampathkumar et al.^91^	NA
Plasmid psPax2	NA	Addgene 12260
Plasmid pCMV-VSV-G	Stewart et al.^92^	Addgene 8454
BAC Bdnf	BACPAC Resources	RP24-149F11
Fosmid Enhancer	BACPAC Resources	WIBR1-0557J07
Fosmid Bdnf	BACPAC Resources	WIBR1-0841J20
Fosmid Downstream	BACPAC Resources	WIBR1-0166C24

**Software and algorithms**		

Fiji, ImageJ 2.1.0/1.53c	Schindelin et al.^93^	https://imagej.net/software/fiji/
Integrative Genomics Viewer 2.8.6	Robinson et al.^94^	https://software.broadinstitute.org/software/igv/
Graphpad Prism 6.0	NA	https://www.graphpad.com/scientific-software/prism/

### Resource availability

#### Lead contact

Further information and requests for resources and reagents directed to, and will be fulfilled by, Antonella Riccio (a.riccio@ucl.ac.uk).

#### Materials availability

New plasmids generated in this study can be obtained by contacting the lead author. These include: pLV-hU6-Enh^g1^-hUbC-dCas9-KRAB-T2a-puro (Figures 4B–4E and S4D–S4G), pLV-hU6-Enh^g2^-hUbC-dCas9-KRAB-T2a-puro (Figures 4B–4E and S4D–S4G), pLV-hU6-Enh^g1^-hUbC-dCas9-VP64-T2a-puro (Figures 4F–4I), BPK1520-Enh^g1^ (Figures 5 and S5), BPK1520-Enh^g2^ (Figures 5 and S5), pCAG-Bdnf (Figure 6 and Figure S5), pLV-hU6-Enh^g1^-hUbC-dCas9-KRAB-T2a-GFP (Figure S6), pLV-hU6-Enh^g2^-hUbC-dCas9-KRAB-T2a-GFP (Figure S6).

### Experimental model and subject details

#### Animals

All experiments performed in this study were approved by the UK Home Office and were performed under the project license 7813074 held by AR. All animal studies were approved by the Institutional Animal Care and Use Committees at University College London.

#### Cortical neuron progenitor cell culture

Cortical progenitor culture was performed essentially as described.^38^ Cortices were dissected from E12.5 C57BL/6J mouse embryos in dissection buffer (2.5 mM Hepes pH 7.4, 30 mM glucose, 1 mM CaCl_2_, 1 mM MgSO_4_, 4 mM NaHCO_3_, 1X HBSS) supplemented with 1 U/mL Dispase I (Sigma) and 0.6 mg/ml DNase I (Sigma). Dissected cortices were digested in dissociation media (1 mM Hepes pH 7.4, 20 mM glucose, 98 mM Na_2_SO_4,_ 30 mM K_2_SO_4,_ 5.8 mM MgCl_2,_ 0.25 mM CaCl_2,_ 0.001% Phenol red) supplemented with 20 U/mL of papain (Worthington) for 25 min at 37°C. After digestion, cortices were washed, dissociated and plated on Nunc dishes (Thermo Fisher Scientific) or glass coverslips coated with 40 μg/mL poly-D-lysine (Sigma) and 2 μg/mL Laminin (BD Bioscience) in DMEM/F12 medium supplemented with 1× B27, 1× N2, 1 mM glutamine, 1 mM NaHCO_3_ and 10 ng/mL of FGF2 (Thermo Fisher Scientific). Cells were plated more densely for NPC cultures harvested after 2 days *in vitro* (DIV) than for PMN cultures harvested at 7 DIV (90 mm dish for 4C-seq and ChIP: NPC 2.5 × 10^6^ cells, PMN 1 × 10^6^ cells; 6-well plates for qRT-PCR analysis: NPC 3.4 × 10^5^ cells, PMN 1.7 × 10^5^ cells; 24-well plates with glass coverslips for imaging: NPC 5.0 × 10^4^ cells, PMN 2.5 × 10^4^ cells). For PMN cultures, after 2 DIV half of the medium was replaced with Neurobasal medium supplemented with 1× B27, 1 mM glutamine and 200 ng/mL NT-3 (Alomone Labs). After 5 DIV, half of the medium was replaced with Neurobasal medium supplemented with 1× B27, 1 mM glutamine, 200 ng/mL NT-3 (Alomone Labs) and 20 μM 5-Fluoro-2ʹ-deoxyuridine (FdU; Merck). Cells were maintained in 37°C, 5% CO_2_ incubators.

#### Cortical neuron culture

Cortical neurons were dissected from E15.5 C57BL/6J mouse embryos and dissociated as above. Neurons were plated on Nunc dishes (Thermo Fisher Scientific) or glass coverslips coated with 40 μg/mL poly-D-lysine (Sigma) and 2 μg/mL Laminin (BD Bioscience) in MEM supplemented with 10% fetal bovine serum, 5% horse serum, 1 mM glutamine and 1× penicillin-streptomycin. After 2–6 h, culture medium was replaced with Neurobasal medium supplemented with 1× B27, 1 mM glutamine, 1× penicillin-streptomycin and 10 μM FdU (Merck). Cells were cultured at 37°C, 5% CO_2_ for 2–7 days.

### Method details

#### RNA isolation and reverse transcription

For transcriptional inhibition, 50 μM of the RNAPII inhibitor DRB (Merck) was added to culture medium for 1h. RNA was isolated from neuronal cultures using TRIzol (Thermo Fisher Scientific) according to the manufacturer’s instruction. RNA was treated with the TURBO DNA-free kit (Thermo Fisher Scientific) before being reversed-transcribed in a 20 μL reaction volume containing random hexamers, RiboLock RNAse inhibitor and RevertAid (Thermo Fisher Scientific). qRT-PCR reactions (20 μL) contained 10 μL SYBR Select Master Mix (Thermo Fisher Scientific) and 0.25 μM primers (sequences shown in Table S3) and were performed on a BioRad CFX qPCR machine.

#### DNA Fluorescence In Situ Hybridization (FISH)

DNA-FISH experiments were performed as described^53^ with some modifications. Cells were fixed for 10 min in 4% PFA (paraformaldehyde, TAAB) in PBS, followed by permeabilization for 10 min in 0.5% Triton X-100 in PBS. After blocking with PBS+ (PBS plus 0.1% casein, 1% BSA, 0.2% fish skin gelatin) for 1h, coverslips were incubated overnight with primary antibodies in PBS + if necessary. For immuno detection, coverslips were washed in PBS, incubated with appropriate secondary antibodies for 1h in PBS+, and washed in PBS. For DNA-FISH without immunostaining, after the PBS + block, coverslips were washed in PBS and then proceeded directly to post-fixation. Post-fixation in 4% PFA (TAAB) in PBS (10 min) was followed by permeabilization in 0.1M HCl, 0.7% Triton X-100 (10 min, on ice), and by denaturation with 70% formamide in 2× SSC (80°C, 30 min). FISH hybridization with probes was carried out overnight at 42°C. Probes (BAC Bdnf RP24-149F11 for lamina association; fosmid probes for double DNA-FISH (Enhancer WIBR1-0557J07, Bdnf WIBR1-0841J20, Downstream WIBR1-0166C24; BACPAC Resources) were labeled with digoxigenin-dUTP or biotin-dUTP using a nick translation kit (Roche), denatured (95°C, 5 min) and pre-annealed (37°C, 45 min) with Cot-1 DNA and salmon sperm DNA in hydridization buffer (50% formamide, 20% dextran sulfate, 2× SSC, 1 mg/mL BSA) immediately before hybridization. Digoxigenin FISH signals were amplified using sheep anti-digoxigenin fluorescein fab fragments (1:50, Roche 11207741910, RRID:AB_514498) and fluorescein rabbit anti-sheep antibodies (1:100, Vector Labs FI-6000, RRID:AB_2336218); biotin probes were detected using streptavidin-555 (1:1000, Molecular Probes, RRID:AB_2571525). For single FISH experiments, digoxigenin labelling was used; for double DNA-FISH pairs of probes with different labels were mixed immediately prior to addition to the coverslip for hybridization. DNA was counterstained with 4′,6-diamidino-2-phenylindole (DAPI). Coverslips were washed and mounted in ProLong Gold (Thermo Fisher Scientific). Confocal images of neuronal nuclei were acquired using a Leica SPE3 confocal microscope for lamina association, or an SP8 confocal microscope for double DNA-FISH. Images were analyzed using Fiji software. Probe coordinates were identified using the 3D Objects Counter tool on hyperstacks of individual nuclei (ensuring only 1 or 2 foci per cell). For double DNA-FISH analysis, the separation of the probe coordinates (distance AB) from each channel were calculated using the formula:

$$AB=\sqrt{{\left(x2-x1\right)}^{2}+{\left(y2-y1\right)}^{2}+{\left(z2-z1\right)}^{2}}$$

For measurements of probe to nuclear periphery, the distance from the centre of the FISH signal to the closest point of the nuclear edge, identified using DAPI staining, was quantified.

#### Chromatin immunoprecipitation

Chromatin immunoprecipitation (ChIP) experiments were performed as described previously^53^ with some modifications. To crosslink proteins with DNA, the medium was removed from neuronal cultures, and crosslinking buffer (0.1 M NaCl, 1 mM EDTA, 0.5 mM EGTA and 25 mM HEPES-KOH, pH 8.0) containing 1% formaldehyde was added for 10 min at room temperature. The cross-linking reaction was stopped by adding glycine to a final concentration of 125 mM. Cells were rinsed three times with ice-cold PBS containing protease inhibitor cocktail and 1 mM PMSF, collected by scraping and centrifuged at 3,000 rpm at 4°C for 10 minutes. Cell pellets were transferred to 1.5 mL tubes and lysed with 20 cell pellet volumes (CPVs) of buffer 1 (50 mM HEPES-KOH, pH 7.5, 140 mM NaCl, 1 mM EDTA, pH 8.0, 10% glycerol, 0.5% NP-40, 0.25% Triton X-100 and complete protease inhibitor cocktail) for 10 min at 4°C. Nuclei were pelleted by centrifugation at 3,000 rpm for 10 min at 4°C, incubated with 20 CPVs of buffer 2 (200 mM NaCl, 1 mM EDTA, pH 8.0, 0.5 mM EGTA, pH 8.0, 10 mM Tris-HCl, pH 8.0, and complete protease inhibitor cocktail) for 10 min at RT and re-pelleted. 4 CPVs of buffer 3 (1 mM EDTA, pH 8.0, 0.5 mM EGTA, pH 8.0, 10 mM Tris-HCl, pH 8.0, and complete protease inhibitor cocktail) were added to the nuclei, and sonication was carried out by applying 20 pulses, 30 seconds each, at 30 seconds intervals. Insoluble materials were removed by centrifugation at 14000 rpm for 10 min at 4 °C, the supernatant was transferred to a new tube, and the final volume of the nuclear lysate was adjusted to 1 ml by adding buffer 3 supplemented to give 150 mM NaCl, 1% Triton X-100, 0.1% sodium deoxycholate in the final chromatin sample. 50 μL of the 1 mL chromatin samples was saved for an Input, whereas the remaining fraction was incubated with 5 μg Rad21 (Abcam ab992, RRID:AB_2176601) antibody and 50 μL Dynabeads (Thermo Fisher Scientific; washed once) and rotated overnight at 4°C. Beads were pelleted and washed with: low-salt buffer (0.1% SDS, 1% Triton X-100, 2 mM EDTA, 20 mM Tris-HCl, pH 8.0, 150 mM NaCl), high-salt buffer (0.1% SDS, 1% Triton X-100, 2 mM EDTA, 20 mM Tris- HCl, pH 8.0, 500 mM NaCl) and LiCl buffer (0.25 M LiCl, 1% IGEPAL CA630, 1% deoxycholic acid (sodium salt), 1 mM EDTA, 10 mM Tris, pH 8.1) and twice with TE buffer (10 mM Tris-HCl, pH 8.0, 1 mM EDTA). For each wash, beads were incubated for 10 min at 4°C while rotating. Immunoprecipitated DNA was eluted by adding elution buffer (0.1 M NaHCO_3_ pH 8.0, 1% SDS) and incubating at 65°C, 5 min and then rotating at RT for 15 min. Crosslinking was reversed by adding 10 μL 5M NaCl and incubating the samples at 65°C overnight. DNA was purified using PCR purification columns (Qiagen), quantified using the Qubit high sensitivity assay, and subjected to qPCR using the same amount of DNA in immunoprecipitated and input PCRs. Primer sequences are shown in Table S3. The protocadherin HS5 region was used as a positive control^95^ and a region on chromosome 5 was used as a negative control.

#### 4C-seq

4C-seq experiments were performed as described previously.^46^ To crosslink proteins with DNA, the medium was removed from neuronal cultures, and crosslinking buffer (0.1 M NaCl, 1 mM EDTA, 0.5 mM EGTA and 25 mM HEPES-KOH, pH 8.0) containing 1% formaldehyde was added for 10 min at room temperature. The cross-linking reaction was stopped by adding glycine to a final concentration of 125 mM. Cells were rinsed three times with ice-cold PBS containing protease inhibitor cocktail and 1 mM PMSF, collected by scraping and centrifuged at 3,000 rpm at 4°C for 10 minutes. Cell pellets were lysed in 10 mL lysis buffer (10 mM Tris pH 8.0, 10 mM NaCl, 0.2% NP40 supplemented with protease inhibitor cocktail and 1 mM PMSF) on ice for 20 min. Nuclei were collected by centrifugation (1800 rpm, 5 min, 4°C), resuspended in 1.2× DpnII buffer and transferred to Protein LoBind tubes (Eppendorff). SDS was added to 0.3% final concentration and nuclei were incubated 1h at 37°C in thermomixer shaking at 900 rpm (30s on, 30s off). Triton X-100 was added to 2% final concentration and nuclei were incubated 1h 37°C in a thermomixer shaking at 900 rpm (30s on, 30s off). 750 Units of DpnII (NEB) was added and incubated overnight at 37°C in a thermomixer shaking at 900 rpm (30s on, 30s off). The next day, the DpnII buffer was replaced with fresh 1.2× DpnII buffer supplemented with 0.3% SDS and 2% Triton X-100 and another 750 Units of DpnII and incubated overnight at 37°C in thermomixer shaking at 900 rpm (30s on, 30s off). Samples of undigested and DpnII-digested DNA was reverse crosslinked and run on a gel to confirm that most DNA fragments were <3 kb after digestion.

Nuclei were centrifuged (1800 rpm, 3 min) and washed twice with 1× T4 DNA ligase buffer before resuspending in 100 μL 1× T4 DNA ligase buffer with 1600 Units T4 DNA ligase (NEB). *In nucleo* ligation was carried out overnight at 16°C without shaking before confirming that high molecular weight products were obtained. Samples were then reverse crosslinked in the presence of proteinase K overnight at 65°C before phenol:chloroform extraction and ethanol precipitation. DNA was quantified using Qubit high sensitivity assays (Thermo Fisher Scientific). 6–10 μg of DNA was digested with 120 Units Csp6I enzyme (Thermo Fisher Scientific) [3–5 Csp6I digests per sample] overnight at 37°C in thermomixer shaking at 900 rpm (30s on, 30s off). After confirmation that Csp6I-digested products were <3 kb, Csp6I was heat inactivated at 65°C for 20 min before phenol:chloroform extraction and ethanol precipitation. DNA was resuspended in 6 mL total volume to allow proximity ligation by 1600 Units T4 DNA ligase (NEB) overnight at 16°C. Samples were purified by phenol:chloroform extraction and ethanol precipitation, followed by PCR purification columns (Qiagen), before quantitation using with Qubit high sensitivity assays (Thermo Fisher Scientific).

4C-seq libraries were generated using Expand Long Template polymerase (Roche) and primers designed using the 4C-seq primer database^48^ (Table S3). Forward primers were generated with the Illumina p1 sequence

(AATGATACGGCGACCACCGAGATCTACACTCTTTCCCTACACGACGCTCTTCCGATCT),

a two-nucleotide barcode to allow multiplexing of samples, and then the primer sequence. Reverse primers were generated with the Illumina p2 sequence:

CAAGCAGAAGACGGCATACGAGATCGGTCTCGGCATTCCTGCTGAACCGCTCTTCCGATCT).

6-10 PCRs were set up per sample to generate library diversity. PCRs were run using the following program: 3 min 94°C; then 29 cycles of 10s 94°C, 1 min 55°C, 3 min 68°C; then 10 min 68°C. PCR products were purified using the High Pure PCR product purification kit (Roche). Libraries were quantified with Qubit high sensitivity assays, assessed using the Agilent Tapestation, and run on an Illumina MiSeq (MiSeq Reagent Kit v3, 150-cycle). 4C-seq data analysis and normalization was performed as described.^48^

#### CRISPR-Cas9 vectors

Single guide RNAs were designed toward the putative Bdnf enhancer using http://crispr.mit.edu/. The sequences of the guide RNAs that we used throughout this study are (last 3 nucleotides are PAM):

**Table:** 

Enh^g1^	GGATTGTTTGGACTTACTCT
Enh^g2^	GTTTTGTCAAGTGTGGGAGC

The backbones for the BPK1520 vector used to express the guide RNA (U6-BsmBIcassette-Sp-sgRNA^96^ was a gift from Keith Joung (Addgene 65777). Annealed oligos composing the different guide RNAs were cloned into the BsmBI site of U6-BsmBIcassette-Sp-sgRNA. The CRISPRi repressor dCas9-KRAB-MECP2^74^ was a gift from Alejandro Chavez and George Church (Addgene 110821). The backbones for the lentiviral vector pLV hU6-sgRNA hUbC-dCas9-KRAB-T2a-puro and pLV hU6-sgRNA hUbC-dCas9-KRAB-T2a-GFP^69^ were a gift from Charles Gersbach (Addgene 71236, 71237) and the same guides targeting the Bdnf enhancer were cloned into the BsmBI site. To generate pLV hU6-sgRNA hUbC-dCas9-VP64-T2a-puro, we cloned the VP64 sequence from pLV hUbC-VP64 dCas9 VP64-T2A-GFP (Addgene 59791^70^) into the pLV hU6-sgRNA hUbC-dCas9-KRAB-T2a-puro vector (Addgene 71236^69^) using NheI.

Bdnf and control EV overexpression vectors were a gift from Christian Rosenmund.^91^

#### Lentiviral production

10 μg of the transfer vector (e.g. pLV hU6-sgRNA hUbC-dCas9-KRAB-T2a-puro [Empty, or containing Enh^g1^ or Enh^g2^]) was transfected into each 10 cm dish of HEK293T cells together with the packaging vectors psPax2 (7.5 μg; Addgene 12260) and pCMV-VSV-G (5 μg; Addgene 8454) using PEImax (67.5 μg; Polysciences) or Lipofectamine-2000 (50 μL; Thermo Fisher Scientific) in Opti-MEM (Thermo Fisher Scientific). The media was changed after 4h to HEK293T media (DMEM plus 10% fetal bovine serum, 2 mM L-glutamine, 1× penicillin/streptomycin). The media containing viral supernatant was harvested 48 and 72h later. Viral supernatant from all plates was combined, passed through 0.45 μm syringe filters, and concentrated using PEG precipitation or ultracentrifugation. For PEG precipitation, PEG was mixed with the media to 10% final concentration and incubated overnight at 4°C. Samples were centrifuged 2500 rpm, 20 min and the supernatant discarded. For ultracentrifugation, media containing viral particles was ultracentrifuged at 24,000 rpm, 2h, 4°C in a Beckman Optima XPN-80 Ultracentrifuge. The pellets were resuspended in Neurobasal media (Thermo Fisher Scientific) at a 200× concentration.

#### Lentiviral addition to cultured neurons

Lentivirus was added to NPC cultures at DIV1, and half the media was changed at DIV2 as usual. When half of the media was changed at DIV5, the new media was supplemented with 1.0 μg/mL puromycin dihydrochloride (Merck) (final conc on cells 0.5 μg/mL) to select for transduced PMN.

For cortical neuron cultures, media was changed from plating media to neurobasal media 2h after plating, and then lentivirus was added 2h later (all DIV 0). The next day, all the media was changed. Half of the media was changed again at DIV 5, when it was supplemented with 2.0 μg/mL puromycin dihydrochloride (final conc on cells 1.0 μg/mL) to select for transduced neurons.

#### RNA-FISH

2-3h after plating in 24-well plates, mouse cortical neurons were transfected using Optimem containing 375 ng dCas9-KRAB-MECP2 DNA, 125 ng BPK1520 (Non-targeting, or containing guides targeting *Bdnf* ^*Enh170*^), 200 ng pBIRD GFP expression vector, and 0.8 μL Lipofectamine 2000 (Thermo Fisher Scientific). After 2h, the medium was replaced with culture media containing 0.33X B27 (serum starve conditions) with or without 50 mM KCl. Cells were cultured for 48h before fixation and Hybridization Chain Reaction (HCR) RNA-FISH.

HCR probe sets targeting the coding sequences of *Bdnf* (B1 initiator, 20 split-initiator probes) and *Lin7c* (B3 initiator, 30 split-initiator probes) were purchased from Molecular Instruments. Experiments were performed based on the manufacturer’s protocol for mammalian cells.^97^ All reagents and materials used were RNAse-free. In brief, transfected cells were fixed with 4% paraformaldehyde (TAAB) at room temperature for 10 mins followed by permeabilization in 70% ethanol for 3h at 4°C. Cells were washed 2 × 5 mins in 2x SSCT Buffer (2x SSC +0.1% Tween20) and pre-hybridized in Probe Hybridization Buffer for 30 minsat 37°C. Cells were then incubated with 1.2 pmol of each probe overnight at 37°C in a humidified chamber. Excess probes were washed off 4 × 5 min with Probe Wash Buffer at 37°C, followed by 2 × 5 min washes in 5x SSC Buffer at room temperature. Pre-amplification was performed in Amplification buffer for 30 minsat room temperature. 18 pmol of each fluorescent hairpin amplifier (B1h1/B1h2 Alexa Fluor 647 and B3h1/B3h2 Alexa Fluor 594) were snap cooled in separate tubes by heating for 90 sat 95°C in a pre-warmed thermocycler and allowed to cool in the dark for 30 min. After pre-amplification, buffer was removed from cells and replaced with cooled hairpins mixed in Amplification Buffer. To enable quantitative HCR imaging, amplification performed for 45 min in the dark at room temperature. Excess hairpins were washed 5 × 5 min in 5x SSCT Buffer, followed by 10 min incubation in 1 μg/mL DAPI in 1x PBS. Cells were mounted in Pro-Long Gold antifade mountant (#P36930, Thermo Fisher Scientific) and cured overnight. Negative controls without probes and without amplification were captured for each repeat.

Airyscan imaging was performed using a Zeiss LSM900 confocal microscope with a 63× Plan Apochromat objective (NA = 1.4) and Airyscan 2 detector with GaAsp technology. Airyscan optimal settings were used for capture, and images were processed using the Zen Blue 3.4 Airyscan 3D processing module with standard settings. DIC microscopy was also performed on all fields of view captured to verify spot locations within cells. For image analysis, masking using the GFP channel was performed on maximum projections with a single macro for all images. Afterward, RNA spot quantification was performed using batch processing in the FISH-Quant plugin on ImJoy, a hybrid computing platform for deep learning image analysis, with filter sigma = 1.0 and spot detection threshold set at 50.

#### Dendritogenesis assays

Assays were carried out as described previously.^90^ Briefly, 2–3h after plating in 24-well plates, mouse cortical neurons were transfected using Optimem containing 375 ng dCas9-KRAB-MECP2 DNA, 125 ng BPK1520 (Non-targeting, or containing guides targeting *Bdnf* ^*Enh170*^), a GFP expression vector (200 ng pBIRD (Figure 5) or 500 ng pCIG vector (EV or pBdnf); Figure S5) and 0.8–1.5 μL Lipofectamine 2000 (Thermo Fisher Scientific). After 2h, the medium was replaced with culture media containing 0.33X B27 (serum starve conditions) with or without 50 mM KCl. Cells were cultured for 48h followed by immunostaining with anti-GFP (Abcam ab13970, RRID:AB_300798, 1:2000). Coverslips were blinded before images of GFP-transfected non-overlapping neurons were obtained using a Zeiss Axio Imager microscope and analyzed in Fiji. For Sholl analysis we used the Simple Neurite tracer plugin, and then samples were deblinded.

#### Immunofluorescence and clustering analysis

Cells grown on coverslips were washed in PBS and then fixed in 4% PFA (TAAB, 20 min, RT). Cells were washed in PBS (3 times 3 min, RT), permeabilized in 0.3% Triton X-100 in PBS (10 min, RT) and then blocked in 5% goat serum, 5% fetal bovine serum in 1× PBS (1h, RT). Primary antibody incubations took place in a humid chamber at 4°C overnight with the following antibodies: chicken anti-GFP (Abcam ab13970, RRID:AB_300798, 1:2000), mouse anti-mCherry (ab125096, RRID:AB_11133266, 1:1000). Cells were washed in PBS (3 times 3 min, RT) before amplification and detection using goat anti-chicken AlexaFluor-488 (Thermo Fisher Scientific A-11039, RRID:AB_2534096, 1:1000) and donkey anti-mouse AlexaFluor-555 (Thermo Fisher Scientific A-31570, RRID:AB_2536180, 1:1000). Coverslips were washed and mounted in ProLong Gold (Thermo Fisher Scientific). DNA was counterstained with 4′,6-diamidino-2-phenylindole (DAPI). Coverslips were blinded and confocal images of neuronal nuclei were acquired using a Leica SPE3 confocal microscope.

Clustering of neuronal cells was analyzed in Fiji using maximal z projections of the DAPI channel (each image was of a single neuronal cluster and its surrounding cells; if the edge of another cluster was in the image this was removed before processing). After applying a Gaussian blur filter (Sigma 4.0) to even out the signal, we used the ‘Find Maxima’ tool to identify each nucleus. The XY coordinates were inputted into R and used to compute the distance between every point and every other point, before the median per image was calculated and samples were deblinded.

#### LNA transfection

NPC were plated as usual in 6-well plates, and then transfected at DIV5 with 100 nM LNA^Neg^ or LNA^Enh^ in Optimem using 1.5 μL Polyethylenimine (PEImax, Polysciences) and centrifugation (500 xg, 5 min). Media was replaced after 2h with PMN media mixed 1:1 with reserved media from the cells prior to transfection. Cells were harvested at DIV7.

#### *In utero* electroporation

*In utero* intracerebroventricular injections with electroporation were performed essentially as described previously.^38^^,^^53^ E13.5 pregnant mice were anesthetized with isoflurane in oxygen carrier (Abbot Laboratories), and the uterine horns were exposed through a small incision in the ventral peritoneum. Plasmid DNA solution (1.0–1.5 μg/μL), prepared using an Endo-Free plasmid purification kit (Qiagen), was mixed with 50 μM antisense LNA GapmeR (*in vivo*-ready, Qiagen) and 0.05% Fast Green (Sigma) and injected through the uterine wall into the lateral ventricles of the embryos using pulled borosilicate needles and a mouth aspirator (Sigma). Five electrical pulses were applied at 40 V (50-ms duration) across the uterine wall at 950 ms intervals using 3-mm platinum Tweezertrodes (Harvard Apparatus) and an ECM-830 BTX square wave electroporator (Harvard Apparatus). The uterine horns were replaced in the abdominal cavity and the abdomen wall, and the skin was sutured. 48h after surgery, pregnant mice were sacrificed, and embryos were subjected to immunofluorescence to assess radial migration.

Embryonic brains were fixed using 4% PFA in PBS overnight at 4°C. Fixed samples were cryoprotected using 30% sucrose overnight at 4°C. Brains were frozen in Optimal Cutting Temperature (OCT, Sakura) and 12 μm coronal sections were cut using a Leica cryostat. Tissue sections were permeabilized in 0.3% Triton X-100, 10% normal goat serum, 2% BSA in PBS at room temperature for 1h and incubated with chicken anti-GFP (Abcam ab13970, RRID:AB_300798, 1:1000) primary antibodies overnight at 4°C. After three sequential washes with PBS, sections were incubated with goat anti-chicken AlexaFluor-488 (Thermo Fisher Scientific A-11039, RRID:AB_2534096, 1:1000) and 4′,6-diamidino-2-phenylindole (DAPI) for 90 min at RT. Sections were washed with PBS and mounted using Fluoromount-G (Southern Biotechnology). Images were acquired on Leica SP8 confocal microscope at 1024 × 1024 pixel resolution; migration analysis images were acquired with a 20× objective, circularity analysis images were acquired with a 63× objective.

### Quantification and Statistical Analysis

All statistics analysis was conducted in GraphPad. In Figures, ∗p<0.05, ∗∗p<0.01, ∗∗∗p<0.001,∗∗∗∗p<0.0001. Details of statistical tests arranged by Figure is below.

#### Figure 1

B) Bars represent mean ± standard error of the mean (SEM); points show results from different biological replicates. Unpaired t-test (two-tailed): Nestin p = 0.0005, *t* = 4.707, n = 7, *df* = 12; Map2 p = 0.0481, *t* = 2.201, n = 7, *df* = 12, NeuN p = 0.0001, *t* = 14.15, n = 3, *df* = 4.

C) Bars represent mean ± SEM; points show results from different biological replicates (n = 7, *df* = 12). Unpaired t-test (two-tailed): Exon I p = 0.0001, *t* = 5.627; Exon II p = 0.0007, *t* = 4.549; Exon III p = 0.0001, *t* = 5.614; Exon IV p = 0.0019, *t* = 3.972; Exon V p = 0.0029, *t* = 3.734; Exon VI p = 0.0021, *t* = 3.899; Exon VIII p = 0.0397, *t* = 2.307; Exon IXa p = 0.0023, *t* = 3.846; Lin7c p = 0.0248, *t* = 2.565.

D) Scatter dot plot of the distribution of the distance between *Bdnf* locus and the edge of the DAPI staining. Solid gray lines denote medians. Mann-Whitney test (two-tailed): p = 0.0002; median of NPC 0.5404, n = 133 (across 4 biological replicates), median of PMN 0.8860, n = 123 (across 4 biological replicates).

#### Figure 2

C) Middle panel, scatter dot plot of inter-probe distance measurements in NPC and PMN cells. Solid lines denote medians. n = 87 (Enh/Bdnf-NPC), 98 (Enh/Bdnf-PMN), 78 (Enh/Dnst-NPC), 74 (Enh/Dnst-PMN) foci across 3 biological replicates. One-way ANOVA (p<0.0001, Kruskal-Wallis statistic 21.13, number of groups 4).

Dunn’s multiple comparisons (two-tailed):

• Enh/Bdnf-NPC vs. Enh/Bdnf-PMN: mean rank diff 51.00, p = 0.0023 ∗∗
• Enh/Bdnf-NPC vs. Enh/Down-NPC: mean rank diff −1.699, p>0.9999.
• Enh/Bdnf-NPC vs. Enh/Down-PMN: mean rank diff −6.280, p>0.9999.
• Enh/Bdnf-PMN vs. Enh/Down-NPC: mean rank diff −52.70, p = 0.0022 ∗∗
• Enh/Bdnf-PMN vs. Enh/Dnst-PMN: mean rank diff −57.28, p = 0.0008 ∗∗∗
• Enh/Down-NPC vs. Enh/Down-PMN: mean rank diff −4.581, p>0.9999.

Right panel, co-localization (defined as an inter-probe distance of 225 nm or less) of FISH signals in double DNA-FISH experiments performed in NPCs and PMNs. Bars represent mean ± SEM, and points show results from different biological replicates (n = 3). Fisher exact test (two-tailed):

• Enh/Bdnf-NPC vs. Enh/Bdnf-PMN p = 0.0051 ∗∗
• Enh/Dnst-NPC vs. Enh/Dnst-PMN p = 0.4950
• Enh/Bdnf-NPC vs. Enh/Dnst-NPC p = 0.8729
• Enh/Bdnf-PMN vs. Enh/Dnst-PMN p = 0.0002 ∗∗∗

#### Figure 3

C) Bars represent mean ± SEM, and points show results from different biological replicates (n = 6). Two-way ANOVA:

- β-actin ex-int: NPC vs. PMN 0.8714% total variation, p = 0.4112, Unt vs. DRB 70.68% total variation, p<0.0001.

- Enh-A: NPC vs. PMN 28.82% total variation, p = 0.0005, Unt vs. DRB 32.91% total variation, p = 0.0003.

- Enh-B: NPC vs. PMN 12.27% total variation, p = 0.0349, Unt vs. DRB 35.81% total variation, p = 0.0010.

- −4.0 kb from the *Lin7c* TSS: NPC vs. PMN 3.130% total variation, p = 0.3983, Unt vs. DRB 27.24% total variation, p = 0.0210.

- −2.0 kb from the *Lin7c* TSS: NPC vs. PMN 0.8507% total variation, p = 0.2862, Unt vs. DRB 82.97% total variation, p<0.0001.

Sidak’s multiple comparisons tests shown in table:

**Table:** 

Primer	Comparison	Mean difference	95% confidence interval (CI)	p
β-actin ex-int	NPC Unt vs. DRB	0.9424	0.5956 to 1.289	<0.0001 ∗∗∗∗
	PMN Unt vs. DRB	0.5910	0.2443 to 0.9377	0.0011 ∗∗
	Unt NPC vs. PMN	0.2608	−0.08592 to 0.6075,	0.1610
Enh-A	NPC Unt vs. DRB	0.2514	−0.05151 to 0.5543	0.1137
	PMN Unt vs. DRB	0.5268	0.2239 to 0.8298	0.0009 ∗∗
	Unt NPC vs. PMN	−0.5019	−0.8048 to −0.1989	0.0014 ∗
Enh-B	NPC Unt vs. DRB	0.4716	−0.1540 to 1.097	0.1599
	PMN Unt vs. DRB	0.9430	0.3175 to 1.569	0.0032 ∗∗
	Unt NPC vs. PMN	−0.6498	−1.275 to −0.02431	0.0410 ∗
−4 kb	NPC Unt vs. DRB	0.6364	0.01723 to 1.256	0.0436 ∗
	PMN Unt vs. DRB	0.2726	−0.3466 to 0.8917	0.5010
	Unt NPC vs. PMN	0.02785	−0.5259 to 0.5816	0.9906
−2 kb	NPC Unt vs. DRB	0.8611	0.6258 to 1.096	<0.0001 ∗∗∗∗
	PMN Unt vs. DRB	0.6290	0.3936 to 0.8643	<0.0001 ∗∗∗∗
	Unt NPC vs. PMN	0.04062	−0.1947 to 0.2760	0.8982

#### Figure 4

B) *Bdnf* ^*Enh170*^ eRNA: paired one-way ANOVA *F* = 42.93, p = 0.0027, n = 5.

Dunnett’s multiple comparisons test: Empty vs. Enh^g1^ mean diff 0.3980, 95% CI 0.3312 to 0.4647, p< 0.0001; Empty vs. Enh^g2^ mean diff 0.3658, 95% CI 0.2135 to 0.5180, p = 0.0023.

C) *Bdnf* coding mRNA: paired one-way ANOVA *F* = 15.93, p = 0.0067, n = 5.

Dunnett’s multiple comparisons test: Empty vs. Enh^g1^ mean diff 0.2436, 95% CI 0.1115 to 0.3756, p = 0.0063; Empty vs. Enh^g2^ mean diff 0.2134, 95% CI 0.01223 to 0.4145, p = 0.0417.

D) All *Bdnf* variants: two-way ANOVA with repeated measures. Treatment: 13.35% total variation, p< 0.0001, n = 5.

Sidak’s multiple comparisons in table:

**Table:** 

Variant	Comparison	Mean difference	95% CI	p
Exon I	Empty vs. Enh^g1^	0.1841	−0.07737 to 0.4456	0.2160
	Empty vs. Enh^g2^	0.2224	−0.03906 to 0.4839	0.1103
Exon II	Empty vs. Enh^g1^	0.03372	−0.2277 to 0.2952	0.9483
	Empty vs. Enh^g2^	0.07696	−0.1845 to 0.3384	0.7594
Exon IV	Empty vs. Enh^g1^	0.2329	−0.02854 to 0.4944	0.0900
	Empty vs. Enh^g2^	0.2827	0.02123 to 0.5442	0.0312 ∗
Exon V	Empty vs. Enh^g1^	0.2543	−0.007201 to 0.5157	0.0583
	Empty vs. Enh^g2^	0.3308	0.06930 to 0.5922	0.0097 ∗∗
Exon VI	Empty vs. Enh^g1^	0.2849	0.02345 to 0.5464	0.0297 ∗
	Empty vs. Enh^g2^	0.2853	0.02380 to 0.5467	0.0294 ∗
Exon VIII	Empty vs. Enh^g1^	0.5301	0.2686 to 0.7916	<0.0001 ∗∗∗∗
	Empty vs. Enh^g2^	0.5136	0.2522 to 0.7751	<0.0001 ∗∗∗∗
Exon IXa	Empty vs. Enh^g1^	0.2606	−0.0008302 to 0.5221	0.0509
	Empty vs. Enh^g2^	0.3337	0.07227 to 0.5952	0.0090 ∗∗

E) Lin7c expression: two-way ANOVA with repeated measures. Treatment: 7.34% total variation, p = 0.0569, n = 5.

Sidak’s multiple comparisons in table:

**Table:** 

Variant	Comparison	Mean difference	95% CI	p
mRNA	Empty vs. Enh^g1^	0.001854	−0.3220 to 0.3257	0.9999
	Empty vs. Enh^g2^	0.1015	−0.2223 to 0.4254	0.7169
−2 kb	Empty vs. Enh^g1^	0.1306	−0.1933 to 0.4544	0.5798
	Empty vs. Enh^g2^	0.2609	−0.06291 to 0.5848	0.1309
−4 kb	Empty vs. Enh^g1^	0.4104	0.08654 to 0.7342	0.0112 ∗
	Empty vs. Enh^g2^	0.06380	−0.2600 to 0.3876	0.8758

F) *Bdnf* ^*Enh170*^ eRNA: paired t-test (two-tailed) *t* = 3.915, *df* = 3, p = 0.0296, n = 4.

G) *Bdnf* coding mRNA: paired t-test (two-tailed) *t* = 2.307, df = 3, p = 0.1043, n = 4.

H) All *Bdnf* variants: two-way ANOVA with repeated measures. Enh^g1^ virus: 30.80% total variation, p< 0.0001, n = 4.

Sidak’s multiple comparisons in table:

**Table:** 

Variant	Comparison	Mean difference	95% CI	p
Exon I	Empty vs. Enh^g1^	−0.8014	−1.681 to 0.07798	0.0889
Exon II	Empty vs. Enh^g1^	−0.7617	−1.641 to 0.1177	0.1178
Exon IV	Empty vs. Enh^g1^	−0.7578	−1.637 to 0.1216	0.1211
Exon V	Empty vs. Enh^g1^	−1.014	−1.894 to −0.1347	0.0177 ∗
Exon VI	Empty vs. Enh^g1^	−0.6684	−1.548 to 0.2110	0.2193
Exon VIII	Empty vs. Enh^g1^	−0.6981	−1.577 to 0.1814	0.1812
Exon IXa	Empty vs. Enh^g1^	−0.6531	−1.533 to 0.2263	0.2412

I) *Lin7c* expression: two-way ANOVA with repeated measures. Treatment: 0.4824% total variation, p= 0.7466, n = 4.

Sidak’s multiple comparisons in table:

**Table:** 

Variant	Comparison	Mean difference	95% CI	p
mRNA	Empty vs. Enh^g1^	−0.08046	−0.7171 to 0.5562	0.9781
−2 kb	Empty vs. Enh^g1^	0.1005	−0.5362 to 0.7372	0.9591
−4 kb	Empty vs. Enh^g1^	0.1056	−0.5310 to 0.7423	0.9531

#### Figure 5

A) Line and error bars, mean number of foci ± SEM Each point represents a cell, n = 30 across 3 biological replicates.

Bdnf: one-way ANOVA*F*=20.19, *p*<0.0001

Dunnett's multiple comparisons test:

• NT vs. NT-KCl mean diff 58.93, 95% CI 40.84 to 77.02, p< 0.0001 ∗∗∗∗
• NT vs. Enh^g1^ mean diff 32.30, 95% CI 14.21 to 50.39, p = 0.0001 ∗∗∗
• NT vs. Enh^g2^ mean diff 33.47, 95% CI 15.38 to 51.56, p< 0.0001 ∗∗∗∗

Lin7c: one-way ANOVA F= 2.942, p= 0.0360

Dunnett's multiple comparisons test:

• NT vs. NT-KCl mean diff 36.93, 95% CI 4.476 to 69.39, p= 0.0213 ∗
• NT vs. Enhg1 mean diff 27.63, 95% CI -4.824 to 60.09, p= 0.1132
• NT vs. Enhg2 mean diff 11.03, 95% CI -21.42 to 43.49, p= 0.756

B) Sholl analysis of the dendritic processes of 30 neurons per condition (10 per biological replicate). For each distance point, the mean number of intersections ±SEM is shown.

Control: two-way ANOVA: virus accounts for 0.03693% total variation (p = 0.0170), distance from soma accounts for 59.10% total variation (p<0.0001), interaction accounts for 1.076% variation (p = 0.0369).

Sidak’s multiple comparisons test (at distances with significance):

**Table:** 

Distance from soma (μm)	Comparison	Mean difference	95% CI	p
15	NT + EV vs. Enh^g2^ +EV	−0.3000	−1.451 to 0.8506	>0.9999
	Enh^g2^ +EV vs. Enh^g2^ +BDNF	−1.600	−2.751 to −0.4494	<0.0001 ∗∗∗∗
20	NT + EV vs. Enh^g2^ +EV	−0.8333	−1.984 to 0.3173	0.8589
	Enh^g2^ +EV vs. Enh^g2^ +BDNF	−1.367	−2.517 to −0.2161	0.0024 ∗∗
25	NT + EV vs. Enh^g2^+EV	−0.8000	−1.951 to 0.3506	0.9336
	Enh^g2^ +EV vs. Enh^g2^ +BDNF	−1.167	−2.317 to −0.01606	0.0407 ∗

KCl: two-way ANOVA: virus accounts for 0.2137% total variation (p<0.0001), distance from soma accounts for 53.50% total variation (p<0.0001), interaction accounts for 1.402% variation (p = 0.0004).

Sidak’s multiple comparisons test (at distances with significance):

**Table:** 

Distance from soma (μm)	Comparison	Mean difference	95% CI	p
20	NT + EV vs. Enh^g2^ +EV	1.267	−0.07227 to 2.606	0.1070
	Enh^g2^ +EV vs. Enh^g2^ +BDNF	−1.400	−2.739 to −0.06106	0.0252 ∗
25	NT + EV vs. Enh^g2^ +EV	2.033	0.6944 to 3.372	<0.0001 ∗∗∗∗
	Enh^g2^ +EV vs. Enh^g2^+BDNF	−1.833	−3.172 to −0.4944	<0.0001 ∗∗∗∗
30	NT + EV vs. Enh^g2^ +EV	1.333	−0.005602 to 2.672	0.0532
	Enh^g2^ +EV vs. Enh^g2^ +BDNF	−1.400	−2.739 to −0.06106	0.0252 ∗
40	NT + EV vs. Enh^g2^ +EV	1.800	0.4611 to 3.139	0.0001 ∗∗∗
	Enh^g2^ +EV vs. Enh^g2^ +BDNF	−0.8000	−2.139 to 0.5389	0.9995

#### Figure 6

A) Bars represent mean ± SEM, and points show values of different biological replicates (n = 4). Paired t-test (two-tailed): LNA^Neg^ vs. LNA^Enh^ p = 0.0155, *t* = 7.940, *df* = 2.

C) Data are from 9 to 10 embryos per condition across 3–4 independent experiments. Bars represent mean ± SEM, and points show values of different embryos. Two-way ANOVA: layer 81.48% variation, p<0.0001; treatment 3.704e-014% variation, p>0.9999; interaction 10.00% variation, p< 0.0001.

Tukey’s post test:

**Table:** 

Layer	Comparison	Mean difference	95% CI	p
VZ-SVZ	LNA^Neg^ vs. LNA^Enh^	−4.856	−12.62 to 2.910	0.2994
	LNA^Neg^ vs. LNA^Enh^ + Bdnf	−7.527	−15.09 to 0.03144	0.0512
	LNA^Enh^ vs. LNA^Enh^ + Bdnf	−2.671	−10.44 to 5.094	0.6906
IZ	LNA^Neg^ vs. LNA^Enh^	−16.35	−24.11 to −8.582	<0.0001 ∗∗∗∗
	LNA^Neg^ vs. LNA^Enh^ + Bdnf	−1.696	−9.254 to 5.863	0.8538
	LNA^Enh^ vs. LNA^Enh^ + Bdnf	14.65	6.886 to 22.42	<0.0001 ∗∗∗∗
CP	LNA^Neg^ vs. LNA^Enh^	21.20	13.44 to 28.97	<0.0001 ∗∗∗∗
	LNA^Neg^ vs. LNA^Enh^ + Bdnf	9.223	1.664 to 16.78	0.0127 ∗
	LNA^Enh^ vs. LNA^Enh^ + Bdnf	−11.98	−19.75 to −4.215	0.0012 ∗∗

E) Quantitation of the circularity of cortical plate cells. Data analyzed from the same embryos as B (9–10 embryos per condition across 3–4 independent experiments). Bars represent mean ± SEM, and points show values of different embryos (average of multiple cells per embryo). One-way ANOVA: F = 5.847, p = 0.0080.

Tukey’s post test:

**Table:** 

Comparison	Mean difference	95% CI	p
LNA^Neg^ vs. LNA^Enh^	−0.08193	−0.1636 to −0.0002927	0.0491 ∗
LNA^Neg^ vs. LNA^Enh^ + Bdnf	0.02655	−0.05291 to 0.1060	0.6879
LNA^Enh^ vs. LNA^Enh^ + Bdnf	0.1085	0.02684 to 0.1901	0.0076 ∗∗

#### Figure S1

C) Bars with error bars represent mean ± SEM, and points show results from different biological replicates (n = 3). Two-way ANOVA:

- HS5: Rad21 vs. IgG 85.80% total variation (p<0.0001), NPC vs. PMN 0.9825% total variation (p= 0.4507)

- Neg: Rad21 vs. IgG 42.86% total variation (p = 0.0302), NPC vs. PMN 0.2304% total variation (p = 0.8519)

- Bdnf-CTCF1: Rad21 vs. IgG 55.23% total variation (p = 0.0112), NPC vs. PMN 0.07009% total variation (p = 0.9098)

- Bdnf-CTCF2: Rad21 vs. IgG 93.26% total variation (p<0.0001), NPC vs. PMN 1.068% total variation (p = 0.2355)

- Lin7c-CTCF: Rad21 vs. IgG 70.96% total variation (p = 0.0013), NPC vs. PMN 0.3727% total variation (p = 0.7339)

Sidak’s multiple comparisons test in table:

**Table:** 

Variant	Comparison	Mean difference	95% CI	p
HS5	NPC-Rad21 vs. NPC-IgG	1.780	0.7545 to 2.805	0.0028 ∗∗
	PMN-Rad21 vs. PMN-IgG	2.136	1.111 to 3.161	0.0009 ∗∗∗
Neg	NPC-Rad21 vs. NPC-IgG	0.2640	−0.4000 to 0.9281	0.5199
	PMN-Rad21 vs. PMN-IgG	0.6361	−0.02793 to 1.300	0.0596
Bdnf-1	NPC-Rad21 vs. NPC-IgG	0.3375	−0.1998 to 0.8748	0.2311
	PMN-Rad21 vs. PMN-IgG	0.5713	0.03399 to 1.109	0.0384 ∗
Bdnf-2	NPC-Rad21 vs. NPC-IgG	1.684	1.096 to 2.271	<0.0001 ∗∗∗∗
	PMN-Rad21 vs. PMN-IgG	1.945	1.358 to 2.533	<0.0001 ∗∗∗∗
Lin7c	NPC-Rad21 vs. NPC-IgG	0.4598	−0.03299 to 0.9527	0.0662
	PMN-Rad21 vs. PMN-IgG	0.7742	0.2814 to 1.267	0.0052 ∗∗

#### Figure S2

Middle panel, scatter dot plot of inter-probe distance measurements in NPC (orange) and PMN (blue) cells. Solid lines denote medians. n = 111 (Bdnf/Enh-NPC), 117 (Bdnf/Enh-PMN), 114 (Dnst/Enh-NPC), 126 (Dnst/Enh-PMN) foci across 4 biological replicates. One-way ANOVA (p= 0.0009, Kruskal-Wallis statistic 16.57, number of groups 4).

Dunn’s multiple comparisons (two-tailed).

• Bdnf/Enh-NPC vs. Bdnf/Enh-PMN: mean rank diff 31.69, p = 0.4619
• Bdnf/Enh-NPC vs. Down/Enh-NPC: mean rank diff −16.55, p> 0.9999
• Bdnf/Enh-NPC vs. Down/Enh-PMN: mean rank diff −36.89, p = 0.2168
• Bdnf/Enh-PMN vs. Down/Enh-NPC: mean rank diff −48.24, p = 0.0403 ∗
• Bdnf/Enh-PMN vs. Down/Enh-PMN: mean rank diff −68.58, p = 0.0005 ∗∗∗
• Down/Enh-NPC vs. Down/Enh-PMN: mean rank diff −20.34, p> 0.9999

Right panel, co-localization (defined as an inter-probe distance of 225 nm or less) of FISH signals in double DNA-FISH experiments performed in NPCs and PMNs. Bars represent mean ± SEM, and points show results from different biological replicates (n = 4). Fisher exact test (two-tailed):

• Bdnf/Enh-NPC vs. Bdnf/Enh-PMN p = 0.0123 ∗
• Dnst/Enh-NPC vs. Dnst/Enh-PMN p = 0.3485
• Bdnf/Enh-NPC vs. Dnst/Enh-NPC p = 0.8924
• Bdnf/Enh-PMN vs. Dnst/Enh-PMN p = 0.0002 ∗∗∗

#### Figure S4

A) Bars represent mean ± SEM, and points show values of different biological replicates (*n* = 4). Bdnf coding unpaired t-test (two-tailed):-KCl vs. +KCl *p* = 0.0002, t = 7.940, df = 6. eRNA: unpaired one-way ANOVA p = 0.0026 F= 12.42.

Dunnett’s multiple comparisons test:

- −KCl vs. +KCl mean diff 1.420, 95% CI 0.5729 to 2.267, p = 0.0033 ∗∗
- +KCl vs. DRB mean diff 1.376, 95% CI 0.5292 to 2.223, p = 0.0040 ∗∗

D) eRNA: one-way ANOVA with repeated measures *F* = 17.72, p = 0.0030, *n* = 3.

Dunnett’s multiple comparisons test:

• Empty vs. Enh^g1^ mean diff 0.5928, 95% CI 0.2597 to 0.9260, p = 0.0040.
• Empty vs. Enh^g2 mean^ diff 0.6070, 95% CI 0.2738 to 0.9402, p = 0.0036.

E) Bdnf coding: one-way ANOVA with repeated measures *F* = 0.5209, p = 0.6186, *n* = 3.

Dunnett’s multiple comparisons test: Empty vs. Enh^g1^ mean diff 0.2162, 95% CI -0.4065 to 0.8389, p = 0.5417; Empty vs. Enh^g2^ mean diff 0.1516, 95% CI -0.4711 to 0.7743, p = 0.7224.

F) All *Bdnf* variants: two-way ANOVA with repeated measures. Treatment: 6.649% total variation, p = 0.0025, *n* = 3.

Sidak’s multiple comparisons in table:

**Table:** 

Variant	Comparison	Mean difference	95% CI	p
Exon I	Empty vs. Enh^g1^	0.04174	−0.2740 to 0.3575	0.9429
	Empty vs. Enh^g2^	0.1061	−0.2097 to 0.4219	0.6871
Exon II	Empty vs. Enh^g1^	0.3041	−0.01164 to 0.6199	0.0608
	Empty vs. Enh^g2^	0.3994	0.08358 to 0.7151	0.0110 ∗
Exon IV	Empty vs. Enh^g1^	0.1460	−0.1698 to 0.4618	0.4956
	Empty vs. Enh^g2^	0.1658	−0.1500 to 0.4815	0.4076
Exon V	Empty vs. Enh^g1^	0.3511	0.03535 to 0.6669	0.0270 ∗
	Empty vs. Enh^g2^	0.1281	−0.1877 to 0.4439	0.5807
Exon VI	Empty vs. Enh^g1^	0.1836	−0.1322 to 0.4993	0.3355
	Empty vs. Enh^g2^	0.1982	−0.1176 to 0.5140	0.2825
Exon VIII	Empty vs. Enh^g1^	0.03486	−0.2809 to 0.3506	0.9598
	Empty vs. Enh^g2^	0.003683	−0.3121 to 0.3195	0.9995
Exon IXa	Empty vs. Enh^g1^	0.1158	−0.2000 to 0.4316	0.6402
	Empty vs. Enh^g2^	0.07563	−0.2402 to 0.3914	0.8252

G) *Lin7c* expression: two-way ANOVA with repeated measures. Treatment: 6.881% total variation, p = 0.0422, n = 3.

Sidak’s multiple comparisons in table:

**Table:** 

Variant	Comparison	Mean difference	95% CI	p
mRNA	Empty vs. Enh^g1^	0.1726	−0.2780 to 0.6233	0.5776
	Empty vs. Enh^g2^	0.07850	−0.3721 to 0.5291	0.8888
−2 kb	Empty vs. Enh^g1^	0.1865	−0.2642 to 0.6371	0.5298
	Empty vs. Enh^g2^	0.09698	−0.3536 to 0.5476	0.8362
−4 kb	Empty vs. Enh^g1^	0.4816	0.03101 to 0.9323	0.0361 ∗
	Empty vs. Enh^g2^	0.1730	−0.2776 to 0.6236	0.5764

#### Figure S5

Sholl analysis of the dendritic processes of 30 neurons per condition (10 per biological replicate). For each distance point, the mean number of intersections ±SEM is shown.

Control: Two-way ANOVA: virus accounts for 0.02394% total variation (p = 0.0113), distance from soma accounts for 69.41% total variation (p< 0.0001), interaction accounts for 0.6129% variation (p = 0.8780).

Sidak’s multiple comparisons test (at distances with significance):

**Table:** 

Distance from soma (μm)	Comparison	Mean difference	95% CI	p
45	NT vs. Enh^g1^	−0.1667	−1.455 to 1.122	>0.9999
	NT vs. Enh^g2^	−1.467	−2.755 to −0.1779	0.0053 ∗∗

KCL: Two-way ANOVA: virus accounts for 0.5800% total variation (p<0.0001), distance from soma accounts for 52.98% total variation (p<0.0001), interaction accounts for 1.054% variation (p = 0.4240).

Sidak’s multiple comparisons test (at distances with significance):

**Table:** 

Distance from soma (μm)	Comparison	Mean difference	95% CI	p
20	NT vs. Enh^g1^	2.100	0.1731 to 4.027	0.0120 ∗
	NT vs. Enh^g2^	2.033	0.1065 to 3.960	0.0211 ∗
25	NT vs. Enh^g1^	2.033	0.1065 to 3.960	0.0211 ∗
	NT vs. Enh^g2^	0.9333	−0.9935 to 2.860	>0.9999
70	NT vs. Enh^g1^	2.100	0.1731 to 4.027	0.0120 ∗
	NT vs. Enh^g2^	1.400	−0.5269 to 3.327	0.8809
75	NT vs. Enh^g1^	2.500	0.5731 to 4.427	0.0003 ∗∗∗
	NT vs. Enh^g2^	1.600	−0.3269 to 3.527	0.4417
80	NT vs. Enh^g1^	1.933	0.006477 to 3.860	0.0475 ∗
	NT vs. Enh^g2^	1.733	−0.1935 to 3.660	0.2029
85	NT vs. Enh^g1^	2.167	0.2398 to 4.094	0.0067 ∗∗
	NT vs. Enh^g2^	1.367	−0.5602 to 3.294	0.9260

C) Quantification of the total length of the dendritic processes of neurons analyzed in B. Bars show mean ± SEM; points show each data for each neuron (n = 30) colored by biological replicate (3 biological replicates). two-way ANOVA: KCl accounts for 2.392% total variation, p = 0.0314; virus accounts for 2.862% total variation, p = 0.0626; interaction 6.320% total variation, p = 0.0025.

Sidak’s multiple comparisons in table:

**Table:** 

Variant	Comparison	Mean difference	95% CI	p
Non-targeting	Control vs. KCl	−497.1	−797.0 to −197.2	0.0003 ∗∗∗
Enh^g1^	Control vs. KCl	108.9	−191.0 to 408.8	0.7645
Enh^g2^	Control vs. KCl	−79.24	−379.1 to 220.6	0.8928

#### Figure S6

B) Bars represent means ± SEM. One-way ANOVA (two-tailed) *F* = 6.999, p = 0.0025, n = 14–15 images over 4 biological replicates. Dunnett’s multiple comparisons test:

- Empty vs. Enh^g1^ mean diff −18.98, 95% of CI -31.73 to −6.227, p = 0.0029 ∗∗

- Empty vs. Enh^g2^ mean diff −17.29, 95% of CI -30.26 to −4.318, p = 0.0076 ∗∗

C) Bars represent means ± SEM. One-way ANOVA (two-tailed) *F*= 4.533, p= 0.0150, n = 15–16 images over 4 biological replicates. Dunnett’s multiple comparisons test:

- Empty vs. Enh^g1^+EV mean diff 19.66, 95% of CI 3.223 to 36.10, p = 0.0167 ∗

- Enh^g1^+EV vs. Enh^g1^+Bdnf mean diff 18.19, 95% of CI 1.752 to 34.63, p = 0.0280 ∗

## Data Availability

4C-seq data is available through GEO (accession number GSE190306). Codes were all previously published.
