# Supplementary material for: A novel intergenic enhancer that regulates Bdnf expression in developing cortical neurons
Source: iScience. 2022 Dec 1;26(1):105695. doi: 10.1016/j.isci.2022.105695 (PMC9792897; doi:10.1016/j.isci.2022.105695)
Supplement: Document S1. Figures S1–S6 and Tables S1–S3 [file mmc1.pdf]

iScience, Volume *26*

Supplemental information

A novel intergenic enhancer that regulates *Bdnf* expression in developing cortical neurons

Emily Brookes, Braulio Martinez De La Cruz, Paraskevi Boulasiki, Ho Yu Alan Au, Wazeer Varsally, Christopher Barrington, Suzana Hadjur, and Antonella Riccio

Supplementary Tables

Supplementary Table S1. Published datasets used in this study. Related to Figure 3 and S3.

Data	Cell type	Reference	Accession number
**HiC**	NPC	Bonev et al., 2017[S1]	GSE96107
	CN	Bonev et al., 2017[S1]	GSE96107
**CTCF ChIP-seq**	NPC	Bonev et al., 2017[S1]	GSE96107
	CN	Bonev et al., 2017[S1]	GSE96107
**H3K4me1 ChIP-seq**	CN – KCl	Policarpi et al., 2017[S2]	GSE75191
	CN + KCl	Policarpi et al., 2017[S2]	GSE75191
**H3K27ac ChIP-seq**	CN – KCl	Policarpi et al., 2017[S2]	GSE75191
	CN + KCl	Policarpi et al., 2017[S2]	GSE75191
**CBP ChIP-seq**	CN - Reelin	Telese et al., 2015[S3]	GSE66710
**CREB ChIP-seq**	CN - Reelin	Telese et al., 2015[S3]	GSE66710
**MED23 ChIP-seq**	CN - Reelin	Telese et al., 2015[S3]	GSE66710
**MEF2C ChIP-seq**	CN - Reelin	Telese et al., 2015[S3]	GSE66710
**MEF2 ChIP-seq**	CN - Reelin	Telese et al., 2015[S3]	GSE66710
**TBR1 ChIP-seq**	E15.5 cortex	Notwell et al., 2016[S4]	GSE71384
**JUNB ChIP-seq**	CN – KCl	Malik et al., 2014[S5]	GSE60192
	CN + KCl	Malik et al., 2014[S5]	GSE60192
**FOS ChIP-seq**	CN – KCl	Malik et al., 2014[S5]	GSE60192
	CN + KCl	Malik et al., 2014[S5]	GSE60192
**DNase HS**	Tissues	ENCODE	
**GRO-seq**	CN - Reelin	Telese et al., 2015[S3]	GSE66710
	CN + Reelin	Telese et al., 2015[S3]	GSE66710

Supplementary Table S2. 4C-seq primers. Related to Figure 2 and STAR Methods.

Bait	Primer sequence
**Exon1_F**	CCGGACATCTGCCTAGGATC
**Exon1_R**	CCCTCCTATCCTAAGAATGC
**Enhancer_F**	TGCTACATGTGGTAAAGATC
**Enhancer_R**	GCTTAGCACCTATGCTCAGT

Primers sequences were taken from the mouse database[S6]. The primers were synthesized with sequences for Illumina sequencing:

F:
AATGATACGGCGACCACCGAGATCTACACTCTTTCCCTACACGACGCTCTTCCGATCT
R:
CAAGCAGAAGACGGCATACGAGATCGGTCTCGGCATTCCTGCTGAACCGCTCTTCCGATCT
with a barcode (GG, AC, AG, CC) upstream of the forward primer sequence.

Supplementary Table S3. Primers used for real time PCR analysis of reverse transcription and chromatin immunoprecipitation products. Related to STAR Methods

Name	Sequence (5'-3')	Application
**Bdnf_allRNA_F1**	GGCCCAACGAAGAAAACCAT	qRT-PCR
**Bdnf_allRNA_R1**	GTTTGCGGCATCCAGGTAAT	qRT-PCR
**Bdnf_putENH-A_F**	GGCTGTACACTTCCTCTCCA	qRT-PCR
**Bdnf_putENH-A_R**	GCTTTCCCCATTCCTTGACT	qRT-PCR
**Bdnf_putENH-B_F**	AGCTGTGCCTTATATGTACTTCA	qRT-PCR
**Bdnf_putENH-B_R**	ACCCTGCTTCTCTTGTTCTCT	qRT-PCR
**Bdnf_Universal_R**	GCCTTCATGCAACCGAAGTA	qRT-PCR
**Bdnf_Ex1_F**	TGCATCTGTTGGGGAGACAA	qRT-PCR
**Bdnf_Ex2_F**	CCATTCAGCACCTTGGACAG	qRT-PCR
**Bdnf_Ex3_F**	GGATGCTTCATTGAGCCCAG	qRT-PCR
**Bdnf_Ex4_F**	AGCTGCCTTGATGTTTACTTTGA	qRT-PCR
**Bdnf_Ex5_F**	TTTCTAGCTTTGTGGTGCGG	qRT-PCR
**Bdnf_Ex6_F**	ATCCGAGAGCTTTGTGTGGA	qRT-PCR
**Bdnf_Ex7_F**	CTGAAAGGGTCTGCGGAACT	qRT-PCR
**Bdnf_Ex8_F**	ATCCCAGGAGAAAGGCTGTG	qRT-PCR
**Bdnf_Ex9_F**	TTACAAGCAGATGGGCCACA	qRT-PCR
**Lin7c_Ex3-4_F**	CCCTGAAGTGAGAGCCAATG	qRT-PCR
**Lin7c_Ex3-4_R**	CTGTCAGCAATTCCACCTGG	qRT-PCR
**Lin7c_upstr-2_F**	ACTGAGCTATACCCATGCCC	qRT-PCR
**Lin7c_upstr-2_R**	AGACCAGGGGCCTACTCTAA	qRT-PCR
**Lin7c_upstr-4_F**	CACACCTTGAATCCCAGCAC	qRT-PCR
**Lin7c_upstr-4_R**	TTGCAGCCAAATCCGAAGAC	qRT-PCR
**NeuN_F**	CCAGGCACTGAGGCCAGCACACAGC	qRT-PCR
**NeuN_R**	CTCCGTGGGGTCGGAAGGGTGG	qRT-PCR
**bactin_F**	TCTTTGCAGCTCCTTCGTTG	qRT-PCR (HK)
**bactin_R**	ACGATGGAGGGGAATACAGC	qRT-PCR (HK)

**bactin_ex-int_F**	GATATCGCTGCGCTGGTC	qRT-PCR
**bactin_ex-int_R**	CATCGATCCCCAAGAAAACC	qRT-PCR
**Bdnf_CTCF1_F**	TTTGGTCCCCTCATTGAGCT	ChIP
**Bdnf_CTCF1_R**	TTCTTTGCGGCTTACACCAC	ChIP
**Bdnf_CTCF2_F**	GCTAGGAAGGTAGAGGGTGC	ChIP
**Bdnf_CTCF2_R**	AGCCAAAATTCCGAACCGAG	ChIP
**Lin7c_CTCF1_F**	CATTTGCTGCCAGTGAAGGA	ChIP
**Lin7c_CTCF1_R**	CTGTCAGCAATTCCACCTGG	ChIP
**HS5_F**	GCCATGGAGATTTTCTTTACATGA	ChIP
**HS5_R**	TGGCAGATGGAACCACTTTTTA	ChIP
**Neg_F**	GGACAATTCAACCGAGGAAA	ChIP
**Neg_R**	TGAACTGGTTTGGTGTGCTC	ChIP

Figure S1

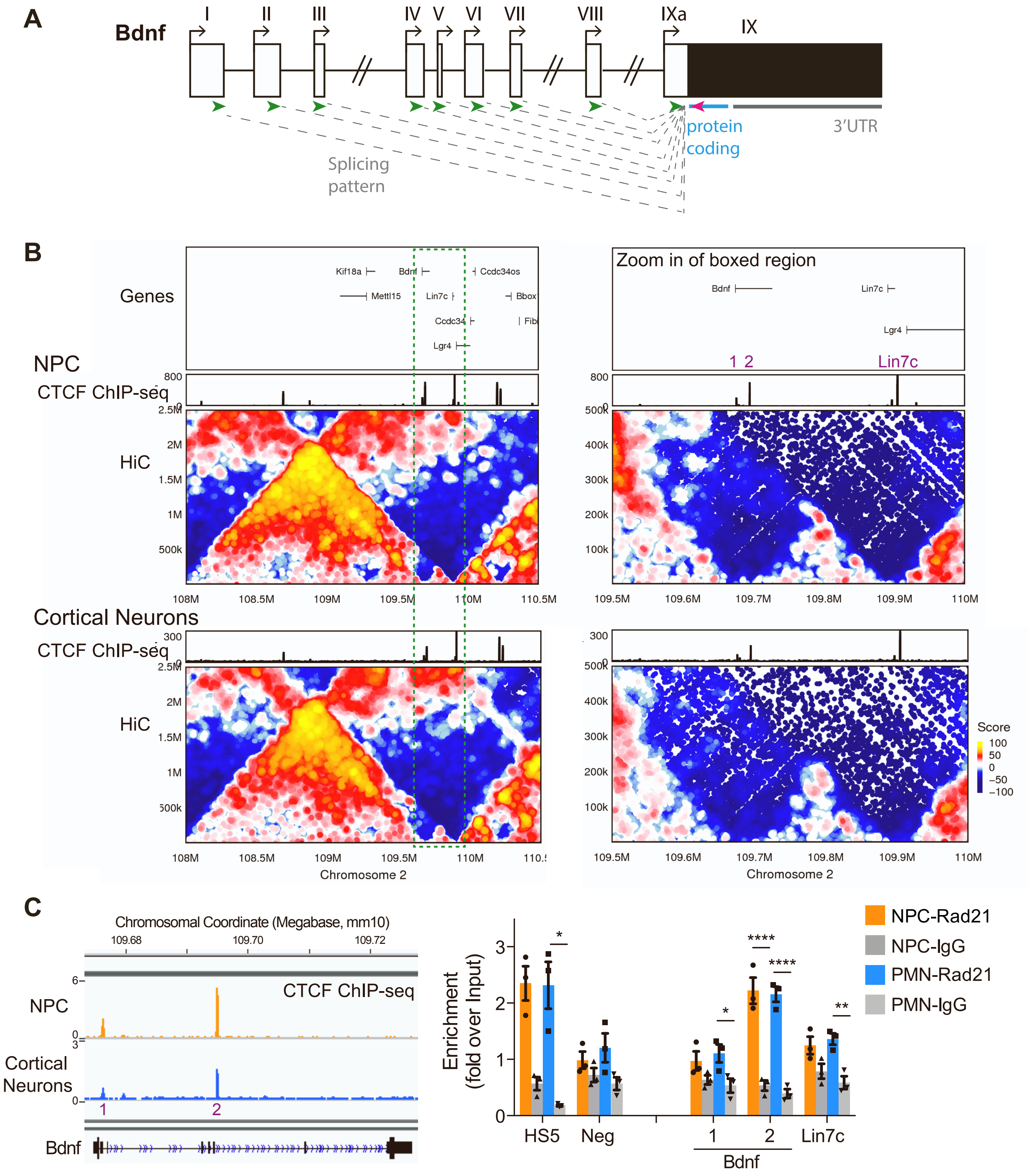

A
Bdnf
I
II
III
IV
V
VI
VII
VIII
IXa
IX
protein
coding
3'UTR
Splicing
pattern
B
Genes
Zoom in of boxed region
NPC
CTCF ChIP-seq
HiC
Cortical Neurons
Score
Chromosome 2
C
Chromosomal Coordinate (Megabase, mm10)
CTCF ChIP-seq
NPC
Cortical
Neurons
Bdnf
Enrichment
(fold over Input)
HS5
Neg
1
2
Lin7c
NPC-Rad21
NPC-IgG
PMN-Rad21
PMN-IgG

Figure S1. *Bdnf* resides in a sub-TAD containing the gene and downstream intergenic region. Related to Figure 1.

A Schematic of the *Bdnf* gene indicating upstream exons (open boxes) which encode alternative 5' untranslated regions (UTRs) that alternatively splice to the universal common exon (IX, black box) which encodes the protein coding sequence (blue) and the 3' UTRs. Arrowheads, primers used to assess variant expression (green=forward, magenta=reverse).

B HiC and CTCF ChIP-seq data from neuronal progenitor cellls (NPCs) and cortical neurons around the *Bdnf* locus discriminates a sub-TAD containing *Bdnf*, a downstream intergenic region and the *Lin7c* gene. This sub-TAD is at the 3' end of a larger TAD, and its boundaries are CTCF-positive. Right panel; zoom of left panel. Purple numbers indicate CTCF-positive sites, which were used for Rad21 ChIP-qPCR.

C Left panel, zoom of CTCF ChIP-seq within Bdnf gene (exons in black). Purple numbers indicate CTCF-positive sites which were used for ChIP-qPCR analysis. ChIP-qPCR of cohesin subunit Rad21 in NPC and PMN confirms binding at *Bdnf* sub-TAD boundaries. Immunoglobulin G (IgG), negative control. The protocadherin HS5 region was used as a positive region for Rad21 binding, and a region on chromosome 5 (Neg) was used as a negative region. Bars with error bars represent mean ±SEM; points show results from biological replicates (n=3). *p<0.05, **p<0.01, ***p<0.001, ****p<0.0001, two-way ANOVA with Sidak's multiple comparisons test (see Methods).

Figure S2

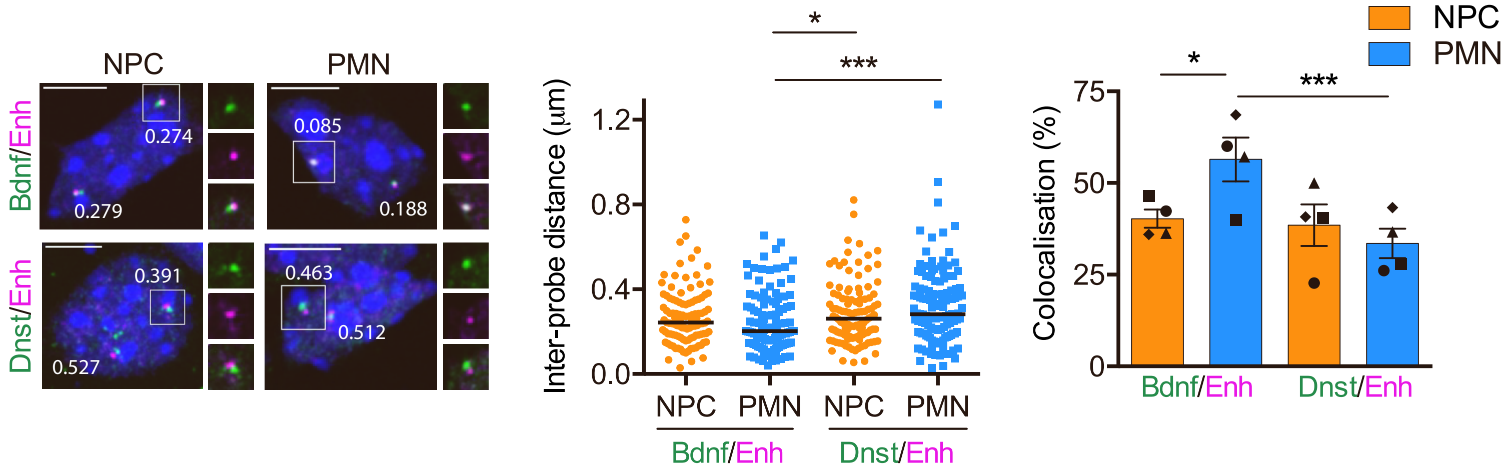

Figure S2. *Bdnf* forms a chromatin loop with an intergenic region and the *Lin7c* gene. Related to Figure 2.
Double DNA-FISH of a probe spanning *Bdnf* ^{Enh170} (Enh) with probes spanning either the *Bdnf* gene or an equidistant region downstream (Dnst) with reciprocal labelling to that shown in Fig. 2C. Probe labelling denoted in coloured font. Left panel, representative images of confocal sections of double DNA-FISH in NPCs and PMNs. Nuclei were stained with DAPI (blue). Middle panel, scatter dot plot of interprobe distance measurements (NPC, orange; PMN, blue). Solid black lines denote means. *n*=111 (Bdnf/Enh-NPC), 117 (Bdnf/Enh-PMN), 114 (Dnst/Enh-NPC), 126 (Dnst/Enh-PMN) foci across 4 biological replicates. **p*<0.05, ****p*<0.001, one-way ANOVA with Dunn's multiple comparisons (two-tailed). Bdnf/Enh-PMN vs. Dnst/Enh-NPC *p*=0.0403; Bdnf/Enh-PMN vs. Dnst/Enh-PMN *p*=0.0005. Right panel, colocalisation (defined as an inter-probe distance of 225nm or less) of FISH signals in double DNA FISH experiments performed in NPCs and PMNs. **p*<0.05, ****p*<0.001, Fisher's exact test (two-tailed). Bdnf/Enh-NPC vs. Bdnf/Enh-PMN *p*=0.0123; Bdnf/Enh-PMN vs. Down/Enh-PMN *p*=0.0002.

Figure S3

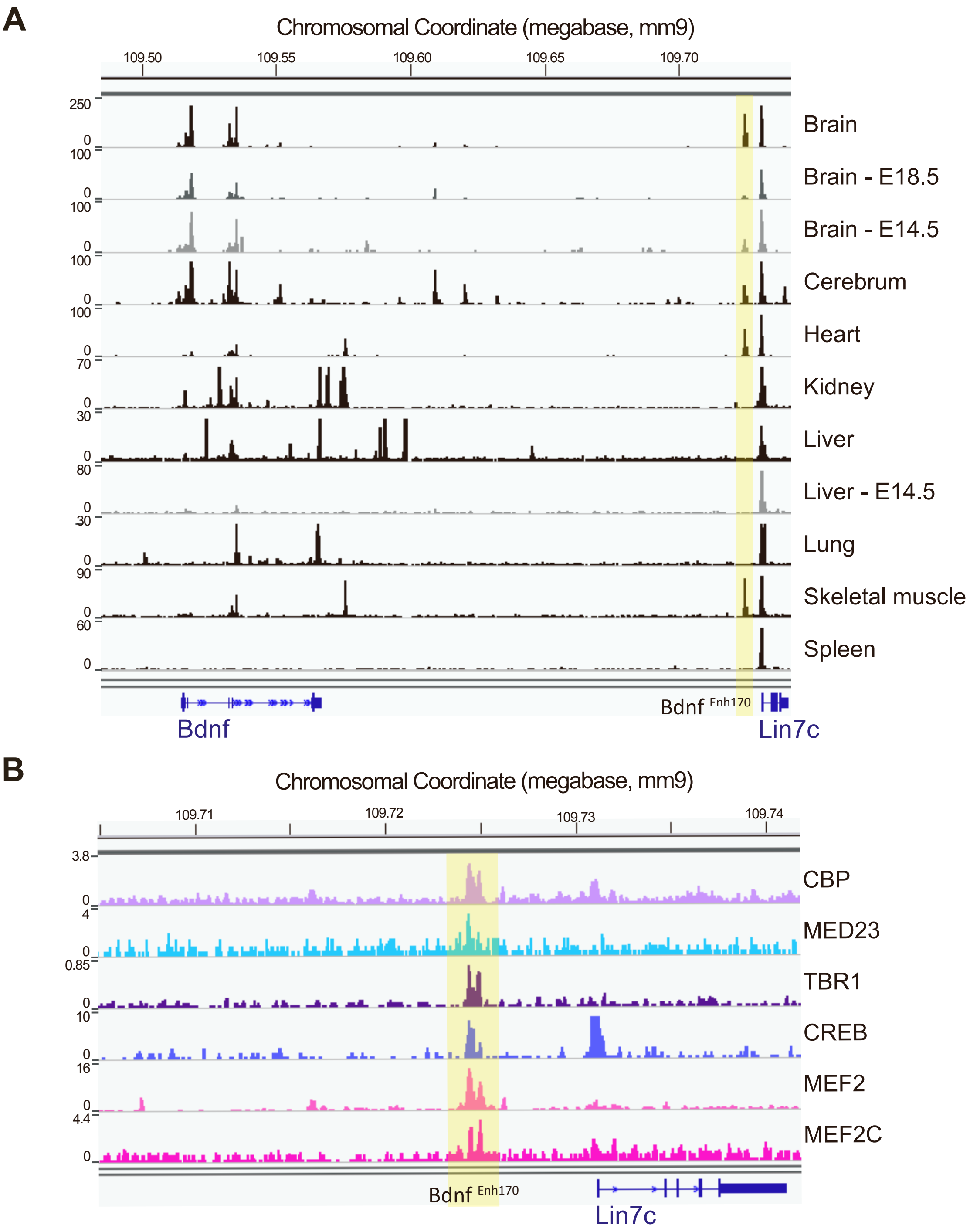

Figure S3. *Bdnf* *Enh170* displays hallmarks of an enhancer. Related to Figure 3.
A DNaseI hypersensitivity across the *Bdnf* locus in the indicated tissues and developmental stages (black, adult; grey, embryonic) demonstrates chromatin accessibility at *Bdnf* *Enh170* and the *Bdnf* gene in embryonic and adult brain and cerebrum, but not in other tissues. ENCODE data mapped to mm9.
B CBP, Mediator subunit MED23, and transcription factors TBR1, CREB, and MEF2 bind to *Bdnf* *Enh170*. Published ChIP-seq datasets (see Supplementary Table S1) mapped to mm9.

Figure S4

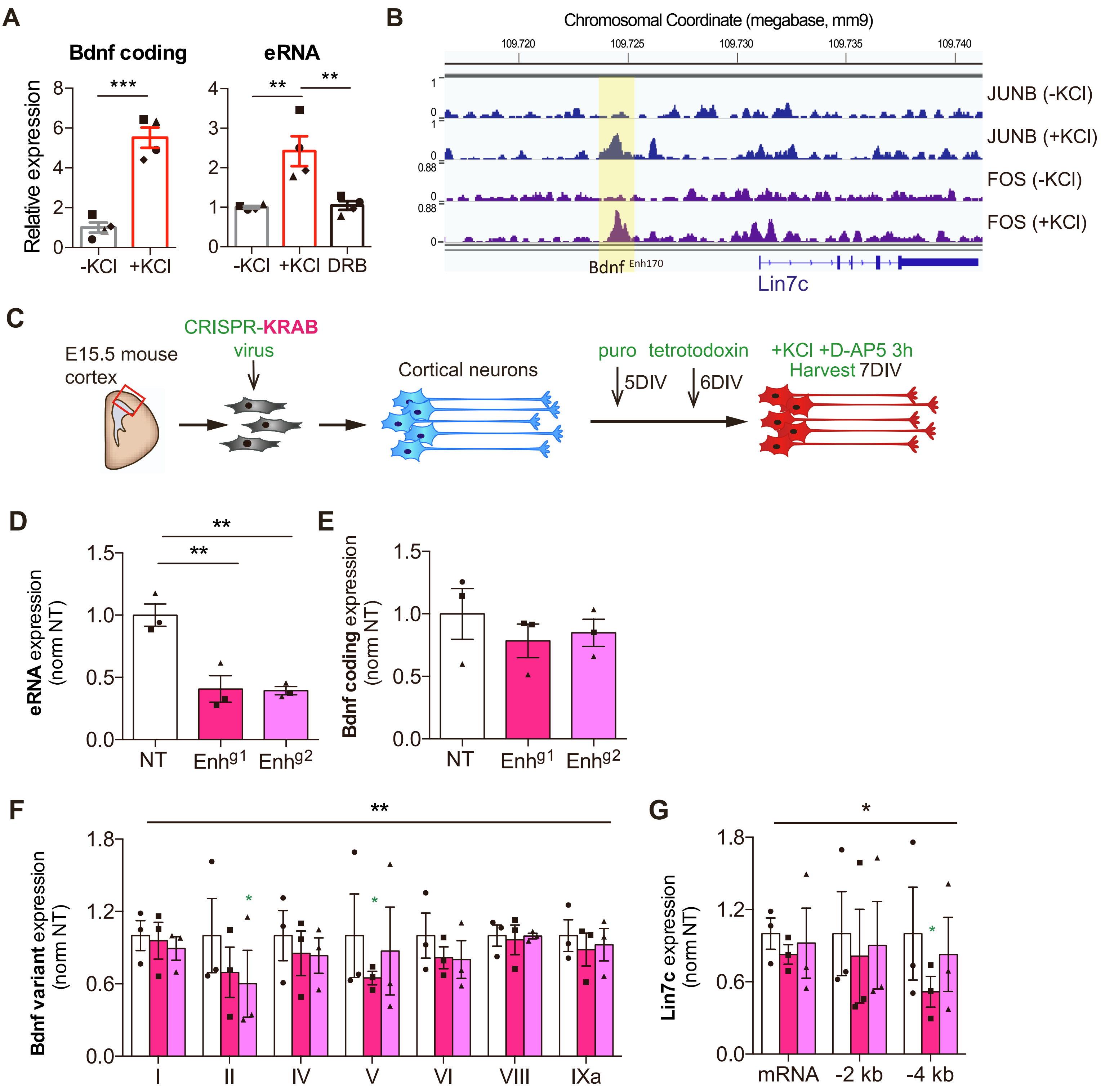
A
Bdnf coding
eRNA
Relative expression
-KCl
+KCl
DRB
B
Chromosomal Coordinate (megabase, mm9)
109.720
109.725
109.730
109.735
109.740
JUNB (-KCl)
JUNB (+KCl)
FOS (-KCl)
FOS (+KCl)
Bdnf Enh170
Lin7c
C
E15.5 mouse cortex
CRISPR-KRAB virus
Cortical neurons
puro
tetrotodoxin
5DIV
6DIV
+KCl +D-AP5 3h
Harvest 7DIV
D
eRNA expression (norm NT)
NT
Enhg1
Enhg2
E
Bdnf coding expression (norm NT)
F
Bdnf variant expression (norm NT)
I
II
IV
V
VI
VIII
IXa
G
Lin7c expression (norm NT)
mRNA
-2 kb
-4 kb

Figure S4. CRISPR inhibition of Bdnf Enh170 reduces activity-dependent Bdnf expression. Related to Figure 5.

A *Bdnf* mRNA and $Bdnf^{Enh170}$ enhancer RNA increase following neuronal depolarization. Expression profile of $Bdnf^{Enh170}$ enhancer RNA and *Bdnf* coding mRNA in cortical neurons maintained in basal (-KCl) or depolarizing (50 mM +KCl) conditions for 48h. Levels assessed by qRT-PCR and normalized to -KCl samples. Bars represent mean ± SEM, and points show values of different biological replicates (*n*=4). ***p*<0.01, ****p*<0.001. *Bdnf* coding, unpaired t-test (two-tailed) *p*=0.0002, *t*=7.940, *df*=6. eRNA, unpaired one-way ANOVA (*p*=0.0026, *F*=12.42) with Dunnett's multiple comparisons test: -KCl vs. +KCl *p*=0.0033, +KCl vs. DRB *p*=0.0040. For full details see Methods.

B AP-1 factors FOS and JUNB ChIP-seq in cortical neurons minus (-) and plus (+) KCl (2h) show activity-dependent recruitment to $Bdnf^{Enh170}$. Published ChIP-seq data (Malik et al., 2014)[S5] mapped to mm9 genome.

C Schematic of CRISPRi virus experiments in depolarized cortical neurons. E15.5, embryonic day 12.5. DIV, days *in vitro*. Puro, puromycin dihydrochloride. D-AP5, D-(-)-2-Amino-5-phosphonopentanoic acid.

D-G qRT-PCR of cortical neurons targeted with lentiviral dCas9-KRAB targeted by no guide (NT, non targeting; open bars) or guides against the enhancer (Enh^{g1}, Enh^{g2}; pink bars) and treated with KCl (25 mM, 3h). Data are normalized to NT-transduced cells. Bars represent mean ± SEM, and points show different biological replicates (*n*=5). **p*<0.05, ***p*<0.01.

D Expression profile of $Bdnf^{Enh170}$ enhancer RNA (eRNA). Paired one-way ANOVA: *F*=17.72, *p*=0.0030, *n*=3. Dunnett's multiple comparisons: Empty vs. Enh^{g1} *p*=0.0040; Empty vs. Enh^{g2} *p*=0.0036.

E Expression profile of Bdnf coding mRNA. Paired one-way ANOVA: *F*=15.93, *p*=0.6186, *n*=3.

F Expression profile of *Bdnf* variants. Two-way ANOVA with Sidak's multiple comparison test (see Methods).

G Expression profile of *Lin7c* variants. Two-way ANOVA with Sidak's multiple comparison test (see Methods)

Figure S5

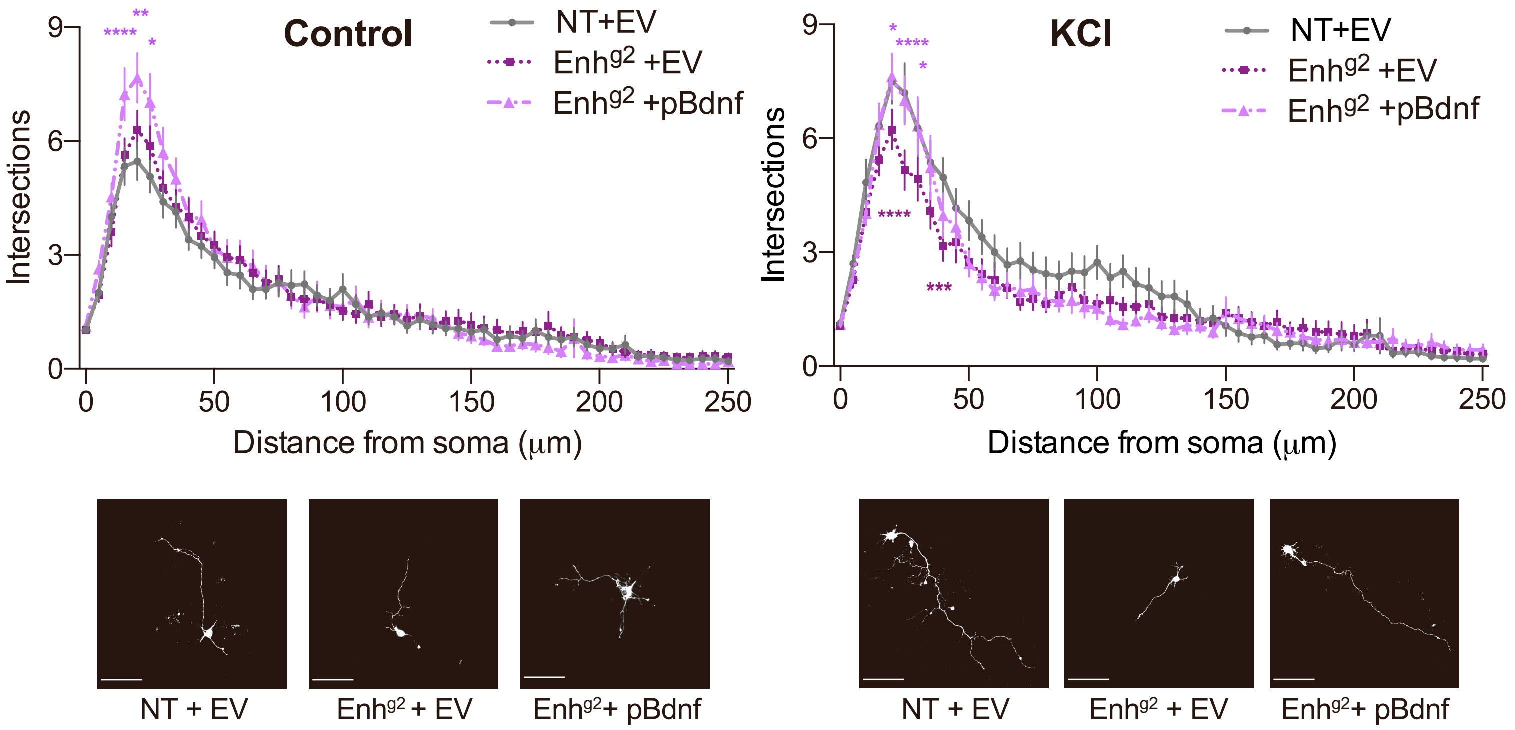

Figure S5. Bdnf expression rescues the effect of *$Bdnf^{Enh170}$* inhibition on activity-dependent dendritogenesis. Related to Figure 5.
Cortical neurons were transfected with a GFP expression vector (Empty vector (EV) or pBdnf) in combination with dCas9-KRAB-MECP2 and an expression vector for guide RNAs (Nontargeting (NT) or targeting the putative enhancer region (Enh^{g2})). Cells were maintained under basal (control) or depolarizing (KCl, 50 mM) conditions for 48 hr, followed by GFP immunostaining. Top, Sholl analysis of the dendritic processes of 30 neurons per treatment (10 per biological replicate). For each distance point, the mean number of intersections ± SEM is shown. **p*<0.05, ***p*<0.01, ****p*<0.001,*****p*<0.0001, two-way ANOVA (Control *p*=0.0170, KCl *p*=0.0004) with Sidak's multiple comparisons test. Control: Enh^{g2} +EV vs. Enh^{g2} +BDNF *p*<0.0001 (15μm), *p*=0.0024 (20μm), *p*=0.0407 (25μm). KCl: NT+EV vs. Enh^{g2} +EV *p*<0.0001 (25μm), *p*=0.0001 (40μm); Enh^{g2} +EV vs. Enh^{g2} +BDNF *p*=0.0252 (20μm), *p*<0.0001 (25μm), *p*=0.0252 (30μm). Lower, representative images. Scale bar, 100 μm.

Figure S6

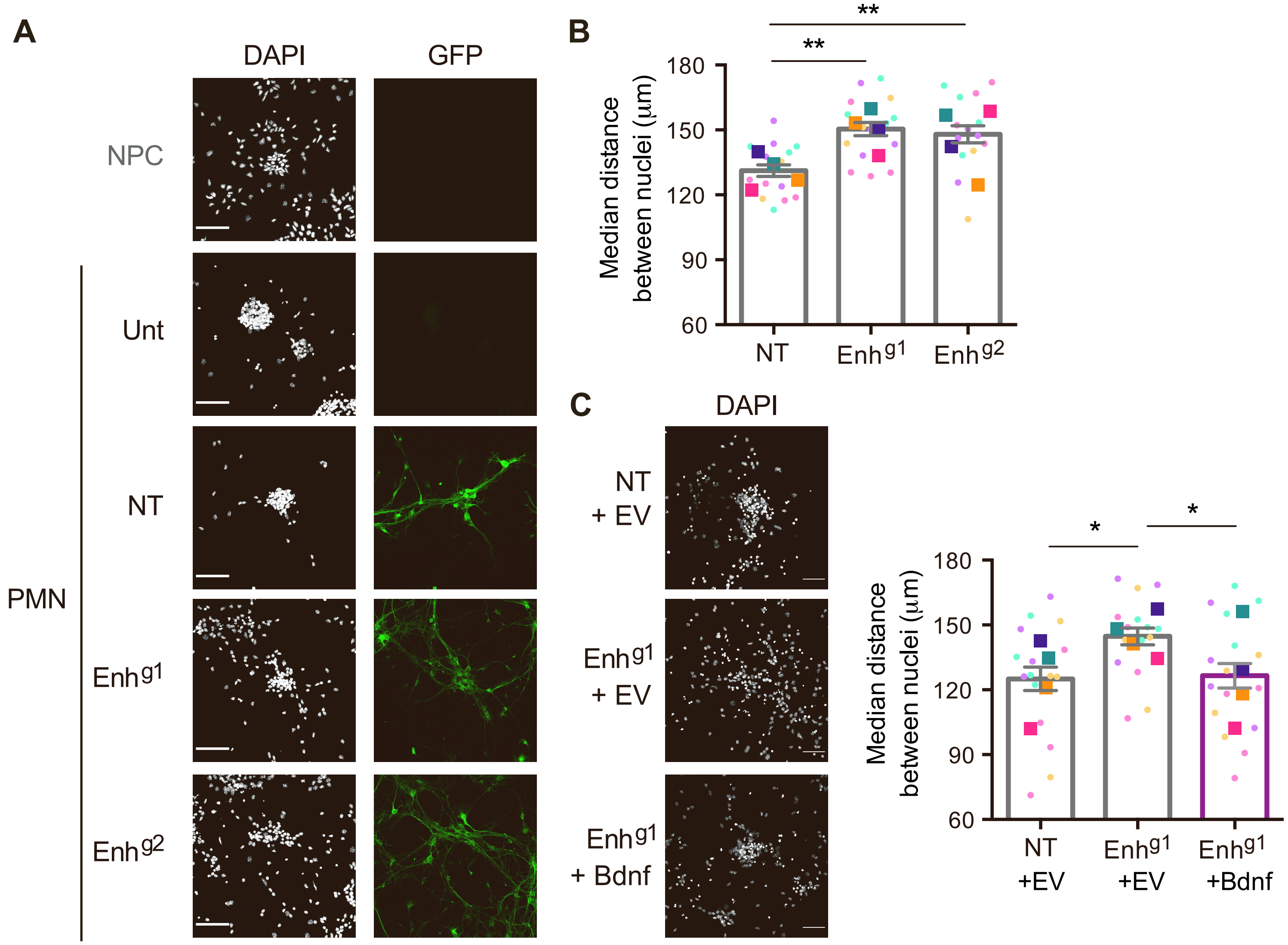

Figure S6. Transcription from *Bdnf* ^{Enh170} is required for neuronal clustering. Related to Figure 6.
A Representative maximal intensity projections of NPC and PMN, and PMN treated with lentiviral CRISPRi (Nontargeting (NT) or targeting the putative enhancer region (Enh^{g1}, Enh^{g2})). DAPI images (grey) show dispersion of cells within clusters; GFP staining (green) shows percentage of lentiviral-targeted cells. Scale bar, 50 µm.
B The median distance between cells per image (image taken to include a single cluster and any surrounding cells) is increased in PMNs when *Bdnf* ^{Enh170} function is compromised. Small dot points show values from individual images colour-coded according to the biological replicate; large square points show means of each biological replicate (*n*=14 over 4 biological replicates (NT), 15 over 4 biological replicates (Enh^{g1}), 14 over 4 biological replicates (Enh^{g2})). Bars represent means ± SEM. **$p<0.01$, unpaired one-way ANOVA (two-tailed, F=6.999, p=0.0025) with Dunnett's multiple comparison test: NT vs. Enh^{g1} p=0.0029; NT vs. Enh^{g2} p=0.0076.
C Expression of Bdnf rescues the increase of cell spacing following enhancer inhibition. Quantification of the median distance between cells per image (image taken to include a single cluster and any surrounding cells) for CRISPRi experiments including control (EV; Empty vector) or Bdnf-expressing lentivirus. Small points show values from individual images colour-coded according to the biological replicate they belong to; large points show means of each biological replicate (n = 16 over 4 biological replicates (NT+EV), 15 over 4 biological replicates (Enh^{g1}+EV), 16 over 4 biological replicates (Enh^{g1}+Bdnf)). Bars represent means ± SEM. *$p<0.05$, unpaired one-way ANOVA (two-tailed, F=4.533, p=0.0150) with Dunnett's multiple comparison test: NT+EV vs. Enh^{g1}+EV p=0.0167; Enh^{g1}+EV vs. Enh^{g1}+Bdnf p=0.0280.

Supplementary References

S1. Bonev, B., Mendelson Cohen, N., Szabo, Q., Fritsch, L., Papadopoulos, G.L., Lubling, Y., Xu, X., Lv, X., Hugnot, J.P., Tanay, A., and Cavalli, G. (2017). Multiscale 3D Genome Rewiring during Mouse Neural Development. Cell *171*, 557-572 e524. 10.1016/j.cell.2017.09.043.

S2. Policarpi, C., Crepaldi, L., Brookes, E., Nitarska, J., French, S.M., Coatti, A., and Riccio, A. (2017). Enhancer SINEs Link Pol III to Pol II Transcription in Neurons. Cell Rep *21*, 2879-2894. 10.1016/j.celrep.2017.11.019.

S3. Telese, F., Ma, Q., Perez, P.M., Notani, D., Oh, S., Li, W., Comoletti, D., Ohgi, K.A., Taylor, H., and Rosenfeld, M.G. (2015). LRP8-Reelin-Regulated Neuronal Enhancer Signature Underlying Learning and Memory Formation. Neuron. 10.1016/j.neuron.2015.03.033.

S4. Notwell, J.H., Heavner, W.E., Darbandi, S.F., Katzman, S., McKenna, W.L., Ortiz-Londono, C.F., Tastad, D., Eckler, M.J., Rubenstein, J.L., McConnell, S.K., et al. (2016). TBR1 regulates autism risk genes in the developing neocortex. Genome Res *26*, 1013-1022. 10.1101/gr.203612.115.

S5. Malik, A.N., Vierbuchen, T., Hemberg, M., Rubin, A.A., Ling, E., Couch, C.H., Stroud, H., Spiegel, I., Farh, K.K., Harmin, D.A., and Greenberg, M.E. (2014). Genome-wide identification and characterization of functional neuronal activity-dependent enhancers. Nature neuroscience *17*, 1330-1339. 10.1038/nn.3808.

S6. van de Werken, H.J., Landan, G., Holwerda, S.J., Hoichman, M., Klous, P., Chachik, R., Splinter, E., Valdes-Quezada, C., Oz, Y., Bouwman, B.A., et al. (2012). Robust 4C-seq data analysis to screen for regulatory DNA interactions. Nature methods *9*, 969-972. 10.1038/nmeth.2173.
